# Nucleophilic dearomatization of 4-aza-6-nitrobenzofuroxan by CH acids in the synthesis of pharmacology-oriented compounds

**DOI:** 10.3762/bjoc.13.277

**Published:** 2017-12-21

**Authors:** Alexey M Starosotnikov, Dmitry V Shkaev, Maxim A Bastrakov, Ivan V Fedyanin, Svyatoslav A Shevelev, Igor L Dalinger

**Affiliations:** 1 N.D. Zelinsky Institute of Organic Chemistry, Leninsky prosp. 47, Moscow 119991, Russia; 2 A.N. Nesmeyanov Institute of Organoelement Compounds, Vavilova str. 28, Moscow 119991, Russia

**Keywords:** CH acids, dearomatization, 1,4-dihydropyridines, furoxans, nitropyridines

## Abstract

4-Aza-6-nitrobenzofuroxan (ANBF) reacts with 1,3-dicarbonyl compounds and other CH acids to give carbon-bonded 1,4-adducts – 1,4-dihydropyridines fused with furoxan ring. In the case of most acidic β-diketones, which exist mainly in the enol form in polar solvents, the reactions proceed in the absence of any added base emphasizing the highly electrophilic character of ANBF. The resulting compounds combine in one molecule NO-donor furoxan ring along with a pharmacologically important 1,4-dihydropyridine fragment and therefore can be considered as prospective platforms for the design of pharmacology-oriented heterocyclic systems.

## Introduction

**Scheme 1:**
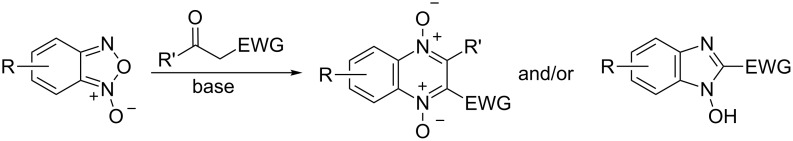
Beirut reaction.

The reactions of condensed furoxans with CH acidic compounds have been extensively studied. There are two main possibilities for these reactions to proceed depending on the structure of the substrate and the nucleophile. The first, Beirut reaction [1–10], allowing to transform the furoxan ring to pyrazine or imidazole *N*-oxides as well as *N*-hydroxyimidazole occurs under the action of α-unsubstituted aldehydes, ketones, 1,3-dicarbonyl compounds, etc. on furoxans annelated with benzene or heterocyclic ring in the presence of base (Scheme 1).

**Scheme 2:**
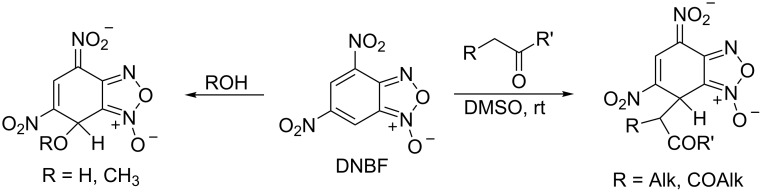
Reactivity of 4,6-dinitrobenzofuroxan.

Mononitrobenzofuroxans as well as pyridofuroxan (4-azabenzofuroxan) react as mentioned above [11–14]. At the same time in the case of highly electrophilic 4,6-dinitrobenzofuroxan (DNBF) another reaction pathway was observed. DNBF react with β-diketones and even monoketones in DMSO solution to give carbon-bonded σ-adducts in the absence of any added base [15] (Scheme 2). However, DNBF is a typical superelectrophile [16], it reacts very easily with water or methanol without base to give the σ-adducts.

**Scheme 3:**
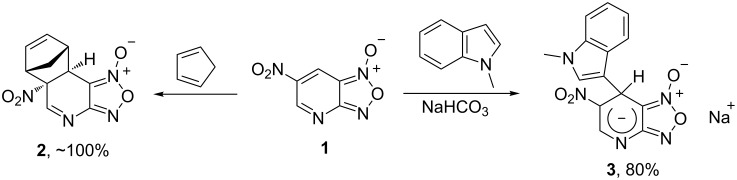
Reactivity of ANBF (**1**).

Earlier it was found that an aza analog of DNBF – 4-aza-6-nitrobenzofuroxan (**1**, ANBF) ranks among the most electrophilic heteroaromatics known to date [17–18]. Compound **1** gives a remarkably stable hydrate in aqueous solution and forms Diels–Alder cycloadduts **2** with dienes (Scheme 3). In addition, ANBF readily forms σ-adduct **3** with *N*-methylindole which was isolated as sodium salt [18] (Scheme 3).

In this work we examined the reactivity of ANBF toward CH-acidic compounds containing a variety of functional groups. This allows to synthesize highly functionalized heteroaromatics combining several pharmacophoric moieties in the same molecule, interesting from the standpoint of design of novel pharmaceuticals. The work continues our ongoing project on the synthesis of complex hybrid molecules – furoxan-containing potential NO donors [19–24].

## Results and Discussion

**Scheme 4:**
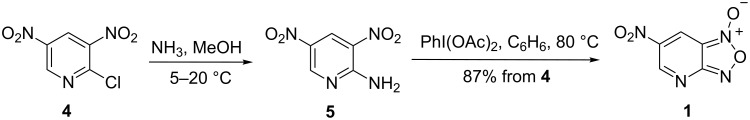
Synthesis of ANBF.

The only method for the synthesis of **1** described so far deals with the reaction of commercially available 2-chloro-3,5-dinitropyridine (**4**) with NaN_3_ followed by thermolysis of the intermediate azide [25–26]. We developed an alternative safe and efficient method for the synthesis of **1** starting from chloride **4** which was treated with methanolic ammonia solution to give 2-amino-3,5-dinitropyridine (**5**). Oxidative cyclization of **5** under the action of PhI(OAc)_2_ gave ANBF in 87% overall yield (Scheme 4).

**Scheme 5:**
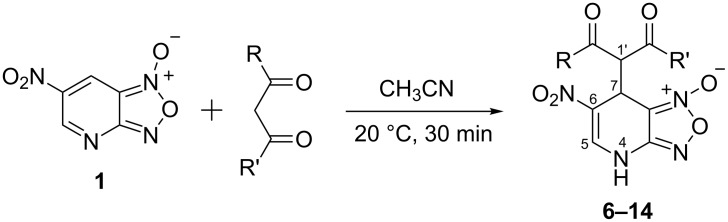
Reactions of ANBF with β-dicarbonyl compounds.

The ^1^H NMR spectra of compound **1** in dry deuterated DMSO and acetone coincided with those described earlier [17,26–27]. Unlike for DNBF [15], no evidence of formation of any adduct was obtained on addition of an equimolar amount of acetone to a solution of ANBF in DMSO-*d*_6_. Moreover, reactions of **1** with acetone or acetophenone in the presence of 1 equiv of Et_3_N led to fast consumption of the starting material and resinification. Apparently, it was caused by interaction of Et_3_N with ANBF because the blank experiment (**1** + Et_3_N with no ketone added) also resulted in decomposition of ANBF. Therefore we studied reactions of **1** with more acidic 1,3-diketones, ketoesters and related compounds. The addition of equimolar quantity of certain CH-acid to the solution of ANBF in dry MeCN or DMF resulted in rapid formation of the adducts **6–14**; TLC analysis showed full consumption of starting materials after 15–20 min stirring at room temperature (Scheme 5, Table 1).

**Table 1:** Reactions of ANBF with β-dicarbonyl compounds.

Entry	CH acid	Products	Isolated yield, %

1	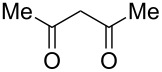	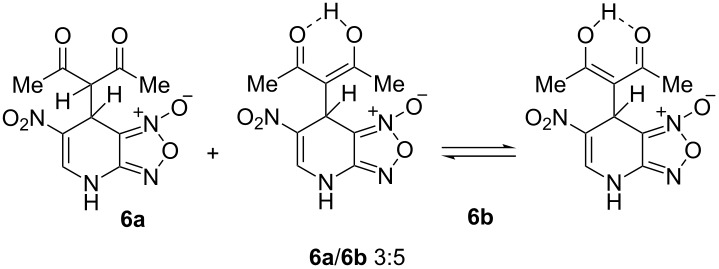	74
2	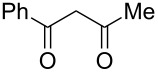	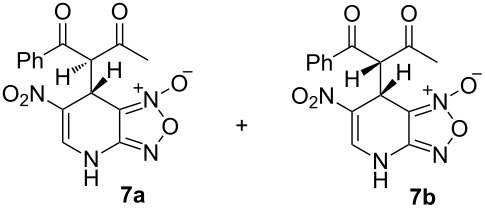	84
3	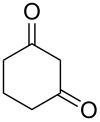	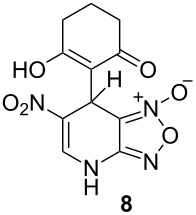	46
4	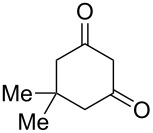	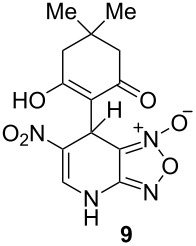	78
5	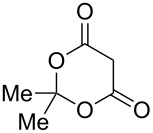	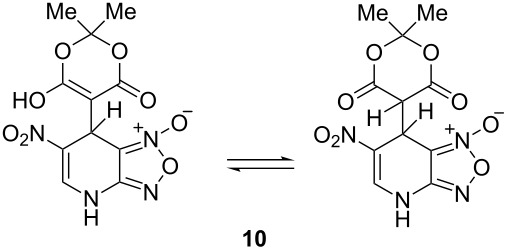	50
6	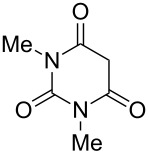	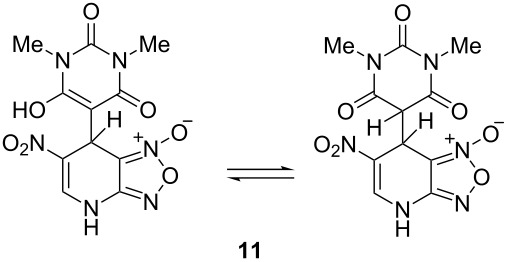	83
7	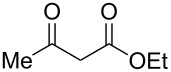	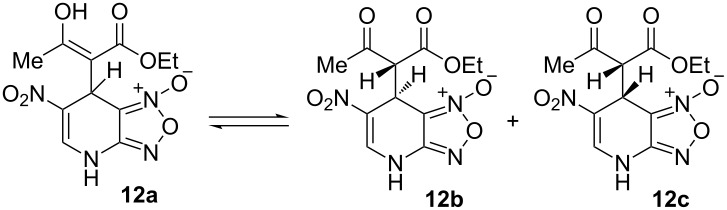	70
8	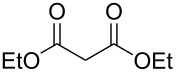	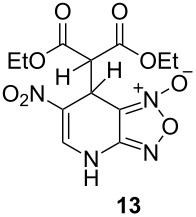	78^a^
9	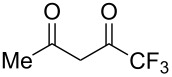	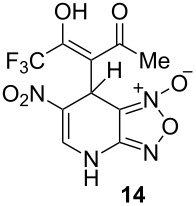	–^b^

^a^Reaction was carried out in the presence of 1 equiv of Et_3_N. ^b^Not isolated due to the low stability of the product.

The reaction with 2,4-pentanedione gave a mixture of diketo-adduct **6a** and a pair of enol tautomers **6b**, NMR spectra in DMSO exhibited two sets of signals with relative intensities about 3:5 at 27 °C. The chirality of carbon atom C7 in compound **6a** makes two methyl groups nonequivalent (δ 2.14 and 2.36 ppm, Table 2). In addition, two doublets at 4.71 and 5.15 ppm (*J* = 2.4 Hz) corresponding to H1’ and H7 atoms were observed. Another set of signals was referred to tautomers **6b**. Due to fast interconversion of these tautomers the signals of their methyl groups represent broad singlets at 2.06 and 2.40 ppm.

**Table 2:** Selected NMR parameters of the ANBF-CH-acid adducts (in DMSO-*d*_6_)^a^.

Adduct	Chemical shifts and coupling constants, δ (*J*)^b^						
	H(5)	H(7)	H(1’)	NH	OH	CH_3_	CH_2_

**6a**	8.41	5.15 (2.4)	4.71 (2.4)	11.72		2.14; 2.36	
**6b**	8.34	5.44		11.72	14.79	2.06; 2.40	
**7a**	8.44	5.59 (2.6)	5.04 (2.6)	11.78		2.17	
**7b**	8.40	5.52 (2.9)	5.34 (2.9)			2.42	
**8**	8.26	5.54		11.47			1.77; 2.33
**9**	8.26	5.52		11.42		0.95	2.23
**13**	8.46	5.05 (3.0)	4.10 (2.9)	12.03		1.10-1.22	3.99–4.17
**14**	8.35	5.38		11.70			2.34

^a^Full spectroscopic data can be found in Supporting Information File 1. ^b^Chemical shifts in ppm from Me_4_Si, *J* values in Hz.

The reaction of ANBF with 1-phenyl-1,3-butanedione resulted in the formation of two diastereomers **7a** and **7b** in a ratio of 6:5 which in DMSO solution exist in diketonic form (Table 1, entry 2). This was confirmed by spectral data: two pairs of dublets (5.04 and 5.59 ppm, *J* = 2.6 Hz, for the major diastereomer and 5.34 and 5.52 ppm, J = 2.9 Hz, for the minor diastereomer) indicate the coupling of H7 and H1’ in both isomers and the absence of the enolic forms (Table 2). However, we were unable to attribute each set of signal to specific diastereomer **7a** or **7b** because the coupling constant values for H7 and H1’ were very close.

1,3-Cyclohexanedione and dimedone gave adducts **8** and **9**, respectively (Table 1, entries 3 and 4) which exist predominantly in the enolic form. In favor of this assertion, in proton NMR spectra of **8** and **9** broad singlets at 2.33 and 2.23 ppm were observed (Table 2), corresponding to methylene groups. At the same time signals of diketonic form were found as traces.

Meldrum’s acid and 1,3-dimethylbarbituric acid react with ANBF similarly in the absence of base to give compounds **10** and **11**, respectively (Table 1, entries 5 and 6). ^1^H NMR spectra of these adducts in DMSO-*d*_6_ contain double sets of signals that may be attributed to dioxo- and enolic forms. However, analysis of their ^1^H and ^13^C NMR spectra was quite complicated due to the broadening of the signals as a result of interconversion of tautomers. On the contrary, ^1^H NMR spectra in acetone-*d*_6_ are much simpler: in each case the single set of signals corresponds to the dioxo form, although the spectrum of **10** contains an additional “enolic” set of signals as traces.

**Figure 1:**
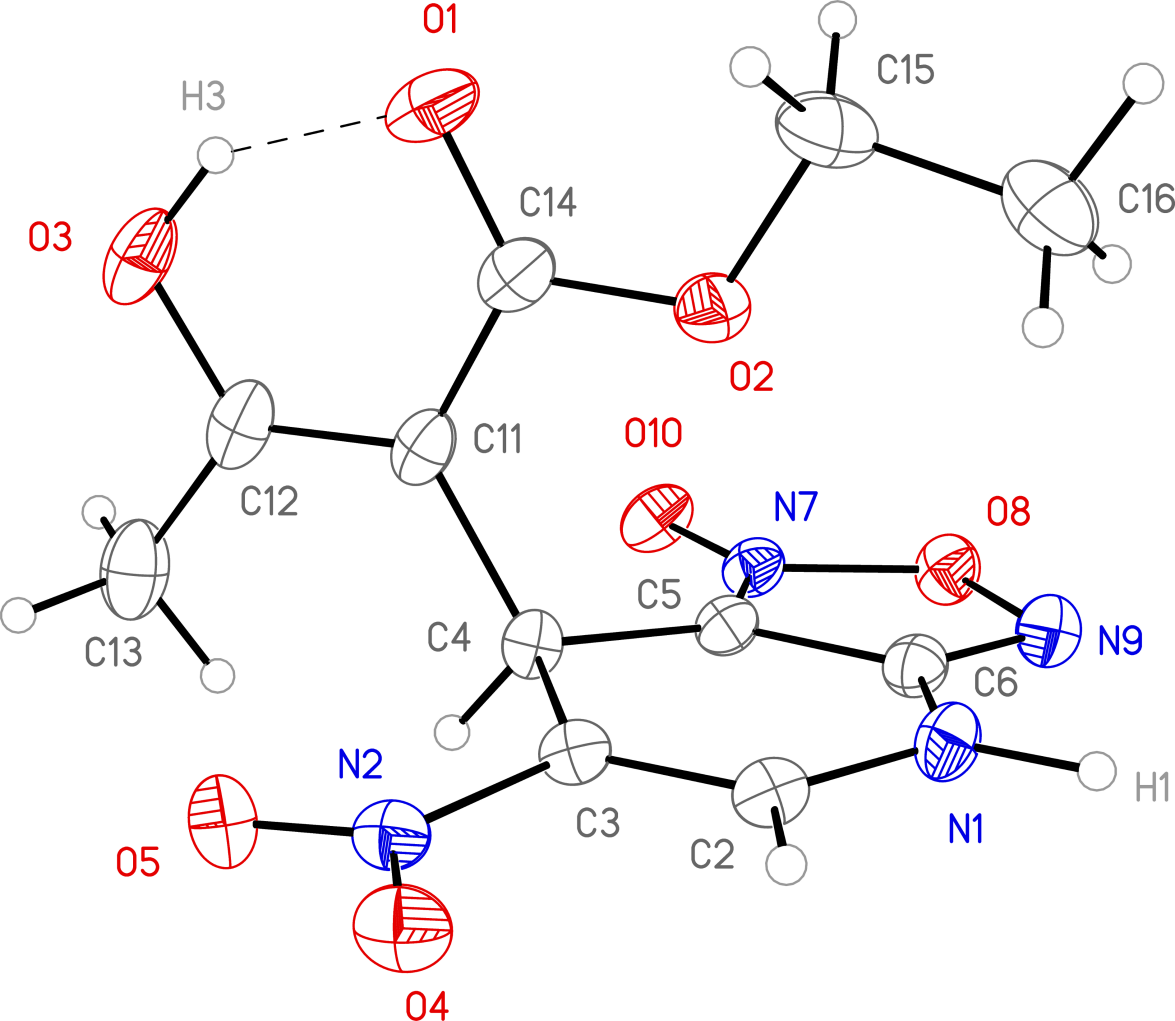
General view of molecule **12** in crystal. Anisotropic displacement parameters for non-hydrogen atoms are drawn at 50% probability; a DMF molecule is omitted for clarity.

Addition of ethyl acetoacetate to the solution of ANBF in acetonitrile resulted in the formation of the corresponding adduct **12** which was isolated in 70% yield (Table 1, entry 7). In this case the product in DMSO solution exists as a mixture of enolic form **12a** and two diastereomeric dicarbonyl compounds **12b** and **12c** in a ratio of 2:1:1. The signals of each form are sometimes overlapping making it difficult to attribute any certain proton. The structure of **12** was confirmed by an X-ray diffraction experiment (Figure 1) [28]. Compound **12** crystallizes as a solvate with one DMF molecule that is connected to the hydrogen atom of the amino fragment by an intermolecular H-bond. In the crystal the position of hydrogen atom H3 (Figure 1), as well as bond lengths (C14–O1 1.2352(17), C12–O3 1.3386(18), C11–C12 1.3687(19), C11–C14 1.450(2) Å) clearly indicate the enolic form of the compound.

The reaction of **1** with less acidic diethyl malonate required the addition of 1 equivalent of Et_3_N. In this case product **13** was isolated in 78% yield (Table 1, entry 8). Again, due to the chirality of C7, the two ethoxy groups are non-equivalent. It causes splitting of the signals of CH_3_, CH_2_ and C=O groups in ^1^H and ^13^C NMR spectra of **13**.

Like all other diketones studied, 1,1,1-trifluoro-2,4-pentanedione gave the corresponding adduct with ANBF (Table 1, entry 9). However, all attempts to isolate compound **14** were unsuccessful due to its instability. Nevertheless, the ^1^H NMR spectrum of crude **14** contains one set of signals which was attributed to the enolic form indicated in Table 2. In particular, the sharp singlet at 5.38 ppm corresponds to the proton H7 of the dihydropyridine ring while the singlet at 2.34 ppm belongs to the methyl group. This is consistent with known data on fluorinated diketones: stability of the enol tautomer increases with the degree of fluorination [29–30]. Apparently this could explain the difference in ratios of tautomers in addition products of structurally similar linear 1,3-diketones (Table 1, entries 1, 2 and 9).

**Scheme 6:**
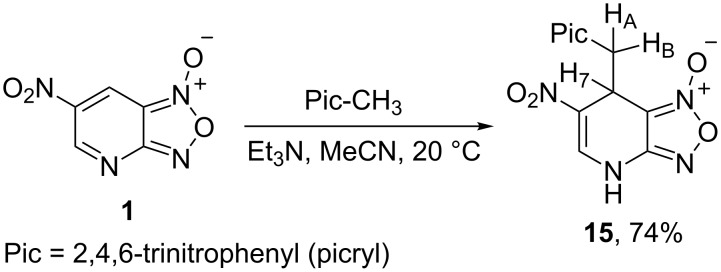
Reaction of ANBF with 2,4,6-trinitrotoluene.

Another class of CH acids was studies in reactions with ANBF. In particular, 2,4-dinitrotoluene in the presence of Et_3_N was found to be unreactive, while 2,4,6-trinitrotoluene (TNT) gave the expected 1,4-adduct **15** in 74% yield (Scheme 6).

**Figure 2:**
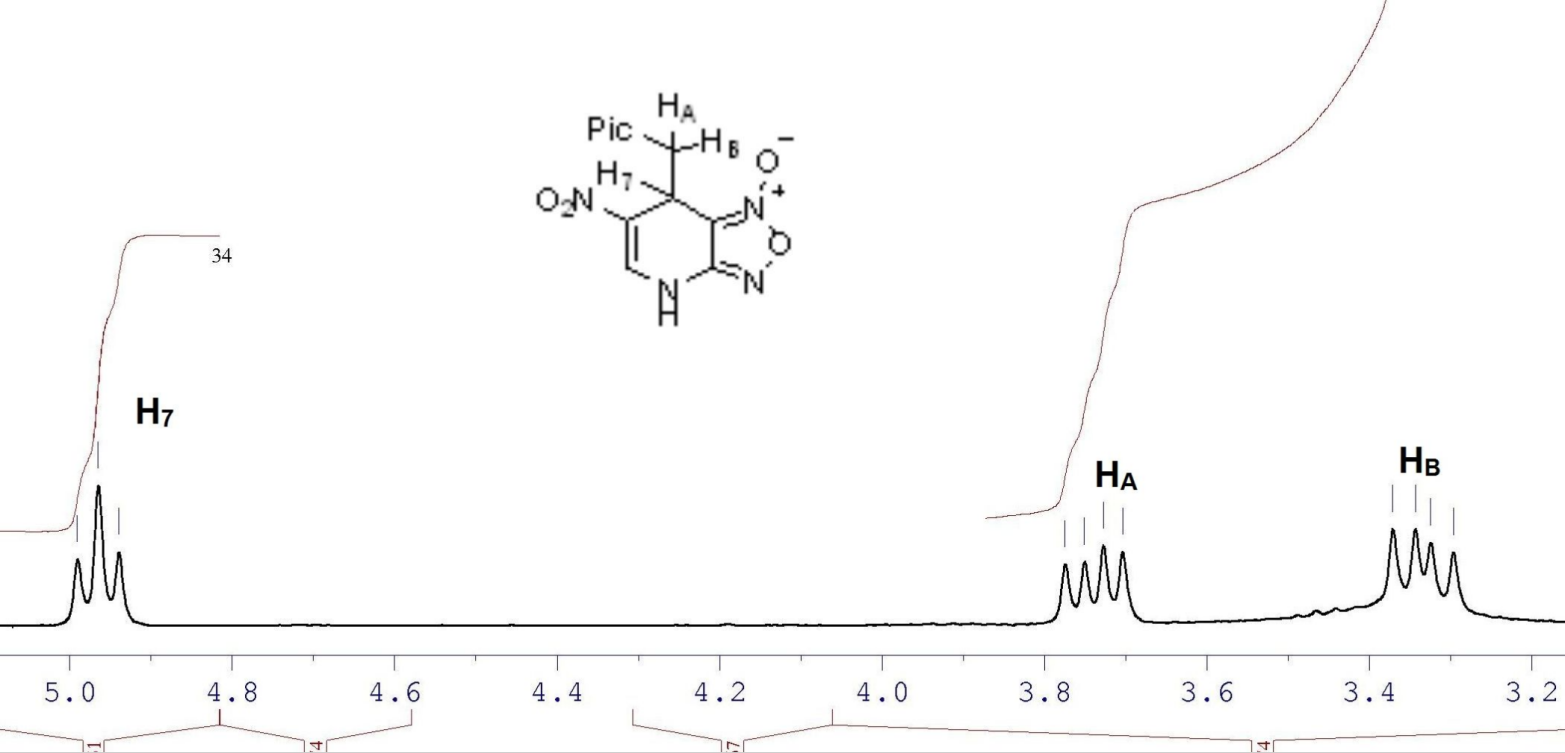
Partial ^1^H NMR spectrum of compound **15** in DMSO-*d*_6_.

Due to the chirality of carbon atom C7, the protons H_A_ and H_B_ of the methylene group are diastereotopic and therefore have different chemical shifts (3.34 and 3.74 ppm). The geminal protons and H7 represent a well-resolved AMX system, Figure 2.

**Figure 3:**
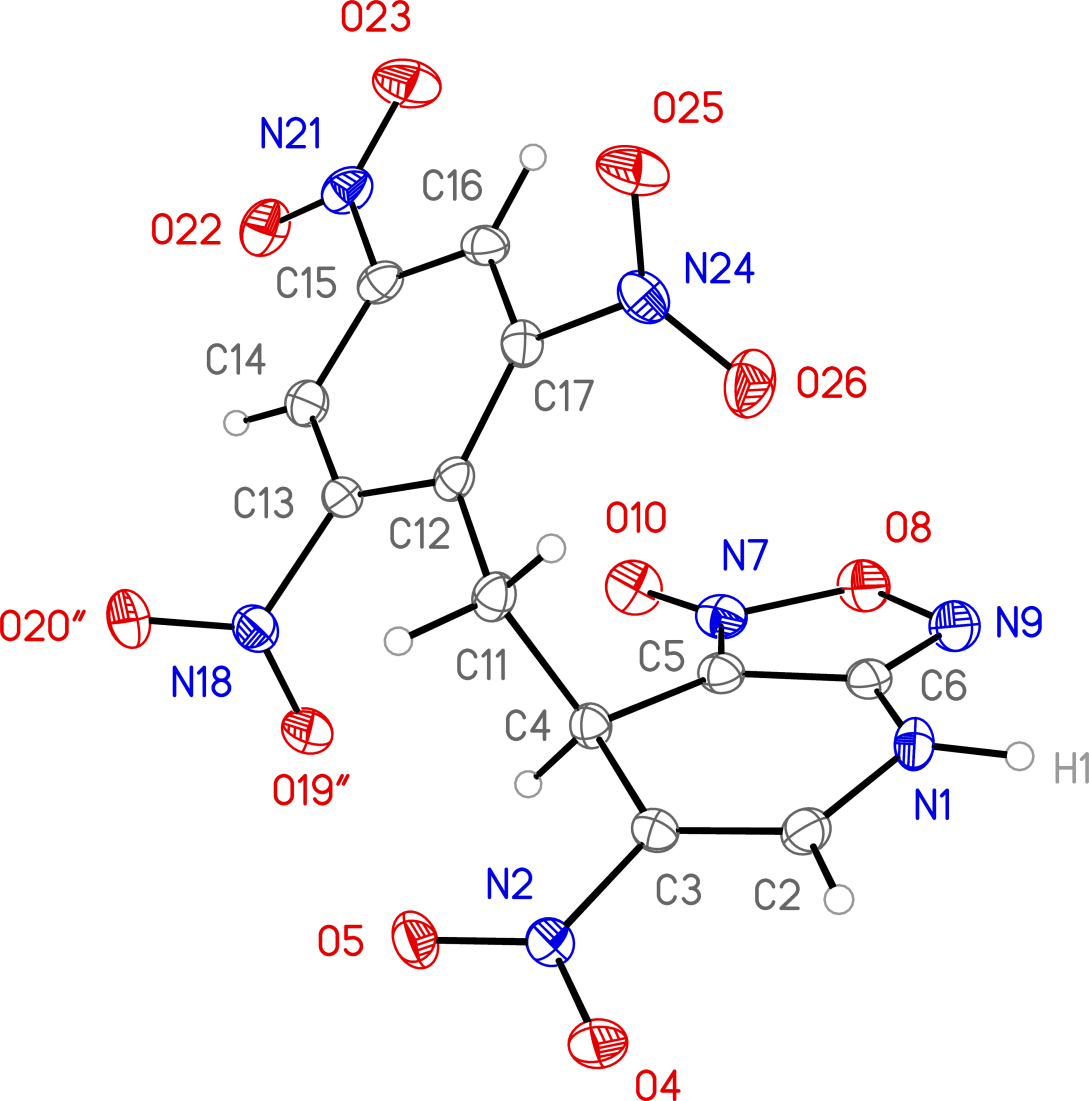
General view of molecule **15** in crystal. Anisotropic displacement parameters for non-hydrogen atoms are drawn at 50% probability; a DMSO molecule and the minor component of the disordered nitro group (N18 O19” O20”) is omitted for clarity.

The structure of compound **15** was additionally studied using X-ray analysis [28] (Figure 3). Compound **15** crystallizes as a solvate with one DMSO molecule connected to the hydrogen atom of the amino fragment by an intermolecular H-bond. Bond lengths and angles in the dearomatized 4-aza-6-nitrobenzofuroxan ring are very similar to those observed in the crystal of **12**·DMF. Due to significant steric effects, the single bond C4–C11 is significantly elongated (1.567(2) Å), and two nitro groups in *ortho*-positions of the connected picryl fragment are rotated out of the plane of the benzene ring.

**Scheme 7:**
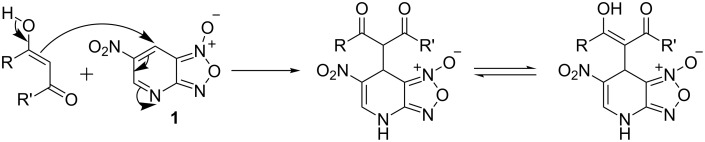
Plausible mechanism of adducts formation.

Thus, reactions of ANBF with strong CH acids (p*K*_a_(H_2_O) 4–11) proceed without added base while in case of diethyl malonate (p*K*_a_(H_2_O) 13.3) and TNT (p*K*_a_(H_2_O) 13.6) the addition of one equivalent of Et_3_N is required. In the latter case the formation of the adducts proceeds through the attack of the anion on C7. All other CH acids used exist mainly in enol form in polar solvents (DMSO, MeCN) and therefore, the mechanism depicted on Scheme 7 seems reasonable.

Adducts of 1,3-dicarbonyl compounds to ANBF are generally enolic. At the same time ratios of enolic and diketonic forms depend on the nature of certain CH acid. In case of most acidic diketones (Table 1, entries 3, 4 and 9) the enol form of the adduct is predominant and the ketonic form was detected by proton NMR as traces. In other cases the products contained comparable quantities of both tautomers, however, enol forms were prevailing. Surprisingly, the benzoylacetone adduct (Table 1, entry 2) represent a mixture of diketonic diastereomers and no enol form was detected.

All compounds synthesized contain two pharmacologically important structural fragments in one molecule, namely dihydropyridine and furoxan rings. The formers are well known L-type calcium channel blockers, used in the treatment of hypertension. Dihydropyridine derivatives are relatively vascular selective in their mechanism of action in lowering blood pressure [31–32]. Dimeric dihydropyridines are used as the precursors for HIV-1 protease inhibitors [33–34]. The furoxan system is used in the design of new NO donors [35–39] and its chemistry is being extensively studied (see for example [40–41]). Additional functionality, such as 1,3-dicarbonyl moiety and nitro group, would provide a number of transformations, heterocyclizations, etc. useful for diversification of synthesized derivatives.

## Conclusion

In conclusion, reactions of 4-aza-6-nitrobenzofuroxan with CH acids gave carbon-bonded 1,4-adducts – 1,4-dihydropyridines fused with furoxan ring. In the case of the most acidic 1,3-dicarbonyl compounds the reactions proceed in the absence of a base. The resulting compounds combine in one molecule the NO-donor furoxan ring along with the pharmacologically important 1,4-dihydropyridine fragment and therefore can be considered as prospective platforms for the design of pharmacology-oriented heterocyclic systems.

## Supporting Information

File 1 Experimental section, NMR spectra, HRMS and X-ray analysis data.
