# Supplementary material for: Nucleophilic dearomatization of 4-aza-6-nitrobenzofuroxan by CH acids in the synthesis of pharmacology-oriented compounds
Source: Beilstein J Org Chem. 2017 Dec 21;13:2854–61. doi: 10.3762/bjoc.13.277 (PMC5753140; doi:10.3762/bjoc.13.277)

Supporting Information

for

Nucleophilic dearomatization of 4-aza-6-nitrobenzofuroxan by CH acids in the synthesis of pharmacology-oriented compounds

Alexey M. Starosotnikov*[1], Dmitry V. Shkaev[1], Maxim A. Bastrakov[1], Ivan V. Fedyanin[2], Svyatoslav A. Shevelev[1] and Igor L. Dalinger[1]

Address: [1]N.D. Zelinsky Institute of Organic Chemistry, Leninsky prosp. 47, Moscow 119991, Russia and [2]A.N. Nesmeyanov Institute of Organoelement Compounds, Vavilova str. 28, Moscow 119991, Russia

Email: Alexey M. Starosotnikov - alexey41@list.ru

*Corresponding author

Experimental section, NMR spectra, HRMS and X-ray analysis data

Experimental

General. All chemicals were of commercial grade and used directly without purification. Melting points were measured on a Stuart SMP 20 apparatus. ^{1}H and ^{13}C NMR spectra were recorded on a Bruker AM-300 spectrometer (at 300.13 and 75.13 MHz, respectively) in DMSO-d_6 or acetone-d_6 with TMS as internal standard. HRMS spectra were recorded on a Bruker micrOTOF II mass spectrometer using ESI. All reactions were monitored by TLC analysis using ALUGRAM SIL G/UV254 plates, which were visualized by UV light.

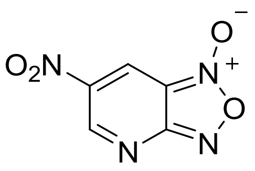

1

4-Aza-6-nitrobenzofuroxan (1)

2-Chloro-3,5-dinitropyridine (22.3 g, 0.11 mol) was added in small portions to a solution of NH_3 in MeOH (7N, 150 mL) at 5–10 °C. The mixture was stirred for 1 h at room temperature and poured in water (500 mL). The resulting solid was filtered, washed with water (100 mL) and dried to give 2-amino-3,5-dinitropyridine (19.55 g, 97%) which was used in next step without further purification.

To a solution of 2-amino-3,5-dinitropyridine (5 g, 27.2 mmol) in benzene (100 mL) $PhI(OAc)_2$ (11.37 g, 35.3 mmol) was added and the mixture was heated under reflux for 5 h. The solvent was evaporated and the residue was washed thoroughly with hexane and ether to give 4-aza-6-nitrobenzofuroxan (**1**, 4.42 g, 90%) as yellow solid. M.p. 95–96 °C (lit. [26] 93–96 °C); ^{1}H NMR (DMSO-d_6, 300 MHz): 9.35 (s, 1H), 9.48 (s, 1H).

Reaction of ANBF 1 with CH acids (general procedure)

To a solution of ANBF (**1**, 0.364g, 2 mmol) in dry CH_3CN (10 mL) the corresponding CH acid (2 mmol) was added and the mixture was stirred for 30 min at room temperature. In

case of compounds **8** and **9** the precipitated product was filtered off and dried on air. In all other cases the mixture was poured in water (50 mL) and filtered or extracted with EtOAc (3 × 30mL) and evaporated to dryness. In case of diethyl malonate and 2,4,6-trinitrotoluene 0.28 mL (2 mmol) of Et_3N was added to the reaction mixture. On completion of the reaction the mixture was poured in water (50 mL), acidified with conc. HCl to pH 2, extracted with EtOAc (3 × 30mL) and evaporated to dryness.

Adduct with acetylacetone: **3-(6-Nitro-1-oxido-4,7-dihydro[1,2,5]oxadiazolo[3,4-*b*]pyridin-7-yl)pentane-2,4-dione monohydrate** (**6a** and **6b**).

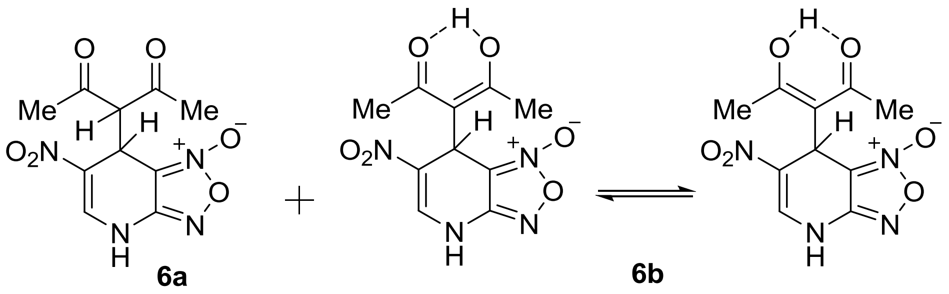

6a:6b - 3:5

M.p. 153-154°C (dec.); ^{1}H NMR (DMSO-d$_6$, 300.13 MHz, δ) for **6a**: 2.14 (s, 3H, CH_3), 2.36 (s, 3H, CH_3), 4.71 (d, J=2.4 Hz, 1H), 5.15 (d, J=2.4 Hz, 1H), 8.41 (s, 1H, H(5)), 11.72 (br.s., 1H, NH); for **6b**: 2.06 (br.s., 3H, CH_3), 2.40 (br.s., 3H, CH_3), 5.44 (s, 1H, H(1')), 8.34 (s, 1H, H(5)), 11.72 (br.s., 1H, NH), 14.79 (br.s., 1H, OH); ^{13}C NMR (DMSO-d$_6$, 75.47 MHz, δ) for mixture of **6a** and **6b**: 29.39, 30.78, 31.07, 31.37, 64.35, 107.21, 123.82, 125.83, 137.73, 139.81, 152.03, 202.62, 204.60 ppm; anal. calcd for $C_{10}H_{12}N_4O_7$ (%): C, 40.01; H, 4.03; N, 18.66; found C, 40.02; H, 3.69; N, 18.76.

Adduct with benzoylacetone: **2-(6-Nitro-1-oxido-4,7-dihydro[1,2,5]oxadiazolo[3,4-*b*]pyridin-7-yl)-1-phenylbutane-1,3-dione** (**7a** and **7b**).

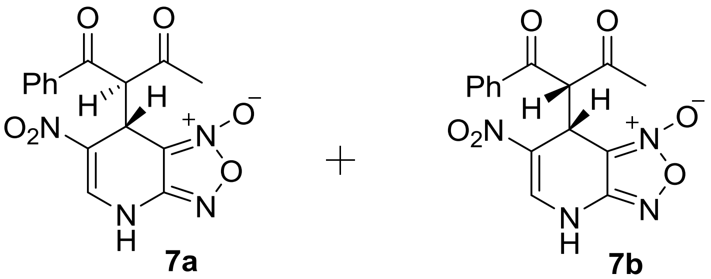

7a + 7b

M.p. 190-191°C (dec); ^{1}H NMR (DMSO-d$_6$, 300.13 MHz, δ): major diastereomer: 2.17 (s, 3H, CH_3), 5.03 (d, J=2.6 Hz, 1H), 5.59 (d, J=2.6 Hz, 1H), 7.48-7.76 (m, 3H), 8.13 (d, J=7.6 Hz, 2H, o-Ph), 8.44 (s, 1H) ppm; minor diastereomer: 2.42 (s, 3H, CH_3), 5.34 (d, J=2.9 Hz, 1H), 5.52 (d, J=2.9 Hz, 1H), 7.48-7.76 (m, 3H), 7.90 (d, J=7.7 Hz, 2H, o-Ph), 8.40 (s, 1H) ppm. HRMS (ESI): $[C_{15}H_{12}N_4O_6 + Na]^+$ calc. 367.0649, found 367.0638. anal. calcd for $C_{15}H_{12}N_4O_6$ (%): C, 52.33; H, 3.51; N, 16.27; found C, 52.31; H, 3.64; N, 16.24.

Adduct with 1,3-cyclohexanedione: **3-Hydroxy-2-(6-nitro-1-oxido-4,7-dihydro[1,2,5]oxadiazolo[3,4-*b*]pyridin-7-yl)cyclohex-2-en-1-one** (**8**).

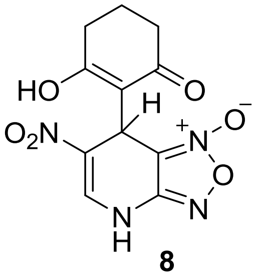

8

M.p. 180-181°C (dec.); ^{1}H NMR (DMSO-d$_6$, 300.13 MHz, δ): 1.75-1.79 (m, 2H, CH_2), 2.33 (br.s., 4H, $2CH_2$), 5.54 (s, 1H, CH), 8.26 (s, 1H, CH), 11.46 (br.s., 2H, NH+OH) ppm; ^{13}C NMR (DMSO-d$_6$, 75.47 MHz, δ): 20.40, 26.47, 29.18, 107.85, 108.53, 125.93, 137.33, 151.85, 217.31 ppm; anal. calcd for $C_{11}H_{10}N_4O_6$ (%): C, 44.90; H, 3.43; N, 19.04; found C, 45.05; H, 3.40; N, 19.12.

Adduct with dimedone: **5,5-Dimethyl-3-hydroxy-2-(6-nitro-1-oxido-4,7-dihydro[1,2,5]oxadiazolo[3,4-*b*]pyridin-7-yl)cyclohex-2-en-1-one** (**9**).

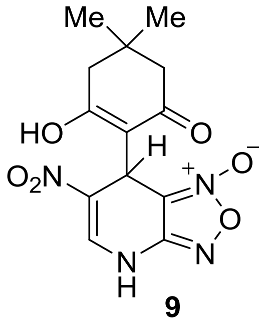

9

M.p. 212-213°C (dec); ^{1}H NMR (DMSO-d_6, 300.13 MHz, δ): 0.95 (s, 6H, $2CH_3$), 2.23 (br.s., 4H, $2CH_2$), 5.52 (s, 1H, CH, H(7)), 8.26 (s, 1H, H(5)), 11.42 (br.s., 2H, NH+OH) ppm; ^{13}C NMR (DMSO-d_6, 75.47 MHz, δ): 27.52, 28.89, 31.58, 107.93, 125.86, 137.31, 151.78, 200.29, 200.95 ppm; HRMS (ESI): $[C_{13}H_{14}N_4O_6 + H]^+$ calc. 323.0986, found 323.0988. anal. calcd for $C_{13}H_{14}N_4O_6$ (%): C, 48.45; H, 4.38; N, 17.38; found C, 48.52; H, 4.33; N, 17.53.

Adduct with Meldrum's acid: **2,2-Dimethyl-5-(6-nitro-1-oxido-4,7-dihydro[1,2,5]oxadiazolo[3,4-*b*]pyridin-7-yl)-1,3-dioxane-4,6-dione** (**10**).

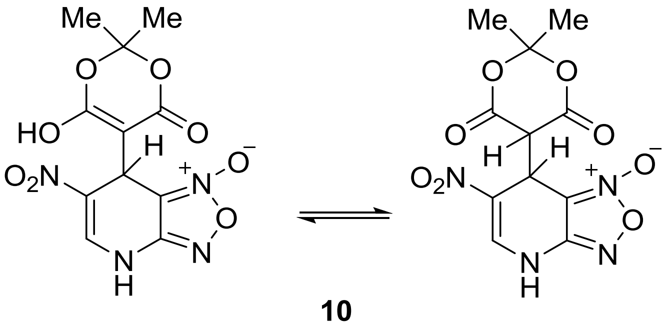

10

M.p. 135-136°C (dec.); ^{1}H NMR (acetone-d_6, 300.13 MHz, δ): 1.75 (s, 3H, CH_3), 1.90 (s, 3H, CH_3), 4.85 (s, 1H, H(1')), 5.64 (s, 1H, H(7)), 8.51 (s, 1H, H(5)), 10.54 (br.s., 1H, NH) ppm. HRMS (ESI): $[C_{11}H_{10}N_4O_8 + H]^+$ calc. 327.0571, found 327.0565. anal. calcd for $C_{11}H_{10}N_4O_8$ (%): C, 40.50; H, 3.09; N, 17.17; found C, 40.32; H, 2.86; N, 17.34.

Adduct with *N*,*N*'-dimethylbarbituric acid: **1,3-Dimethyl-5-(6-nitro-1-oxido-4,7-dihydro[1,2,5]oxadiazolo[3,4-*b*]pyridin-7-yl)pyrimidine-2,4,6(1*H*,3*H*,5*H*)-trione** (**11**).

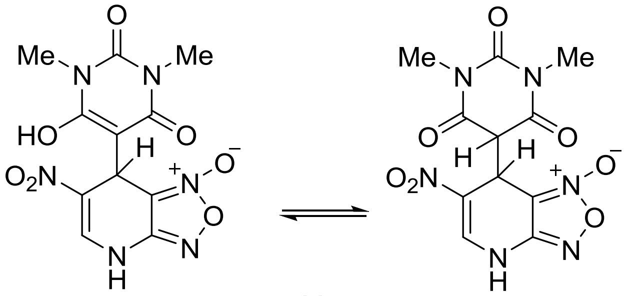

11

M.p. 186-187°C (dec.); ^{1}H NMR (acetone-d$_6$, 300.13 MHz, δ): 3.22 (s, 3H, CH_3), 3.27 (s, 3H, CH_3), 4.51 (s, 1H, H(1')), 5.46 (d, 1H, J=3.0 Hz, H(7)), 8.53 (s, 1H, H(5)), 10.58 (br.s., 1H, NH) ppm; HRMS (ESI): $[C_{11}H_{10}N_6O_7 + H]^+$ calc. 339.0684, found 339.0679. anal. calcd for $C_{11}H_{10}N_6O_7$ (%): C, 39.06; H, 2.98; N, 24.85; found C, 38.94; H, 3.01; N, 24.85.

Adduct with ethyl acetoacetate: **Ethyl 2-(6-nitro-1-oxido-4,7-dihydro[1,2,5]oxadiazolo[3,4-*b*]pyridin-7-yl)-3-oxobutanoate** (**12**).

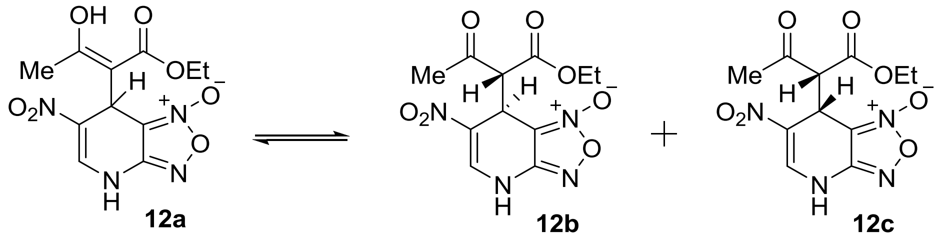

M.p. 143-144°C (dec.); ^{1}H NMR (DMSO-d$_6$, 300.13 MHz, δ): for **12a**: 1.06-1.23 (m, 3H, CH_3), 3.99-4.20 (m, 2H, CH_2), 5.37 (s, 1H, H(7)), 8.37 (s, 1H, H(5)), 11.73 (br.s., 1H, NH), 13.01 (s, 1H, OH) ppm; for **12b+12c**: 1.06-1.23 (m, 3H, CH_3), 3.99-4.20 (m, 2H, CH_2), 4.24 and 4.42 (both d, 1H, J=2.5 Hz, H(1')), 4.98 and 5.2 (both d, 1H, J=2.5 Hz, H(7)), 8.2 and 8.46 (both s, 1H, H(5)), 10.90 and 11.73 (both br.s., 1H, NH) ppm; anal. calcd for $C_{11}H_{12}N_4O_7$ (%): C, 42.31; H, 3.87; N, 17.94; found C, 42.45; H, 4.69; N, 17.73.

Adduct with diethylmalonate: **Diethyl (6-nitro-1-oxido-4,7-dihydro[1,2,5]oxadiazolo[3,4-*b*]pyridin-7-yl)malonate** (**13**).

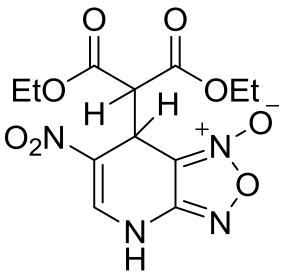

13

Brown oil. ^{1}H NMR (DMSO-d$_6$, 300.13 MHz, δ): 1.10-1.22 (m, 6H, 2CH$_3$), 3.99-4.17 (m, 5H, 2CH$_2$ + CH), 5.05 (d, 1H, J = 3.0 Hz, H(7)), 8.46 (s, 1H, H(5)), 12.03 (br.s, 1H, NH) ppm. ^{13}C NMR (DMSO-d$_6$, 75.47 MHz, δ): 14.08 and 14.36 (2 CH$_3$), 33.08, 51.05, 62.00 and 62.43 (2 CH$_2$), 106.75, 123.45, 140.77, 152.33, 166.24 and 166.97 (2 C=O) ppm; anal. calcd for $C_{12}H_{14}N_4O_8$ (%): C, 42.11; H, 4.12; N, 16.37; found C, 42.35; H, 4.39; N, 16.22.

Adduct with 1,1,1-trifluoroacetylacetone: **(3*Z*)-5,5,5-trifluoro-4-hydroxy-3-(6-nitro-1-oxido-4,7-dihydro[1,2,5]oxadiazolo[3,4-*b*]pyridin-7-yl)pent-3-en-2-one** (**14**).

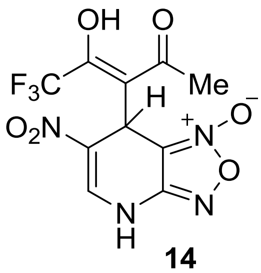

14

Not isolated. ^{1}H NMR (DMSO-d$_6$, 300.13 MHz, δ): 2.34 (br.s., 3H, CH$_3$), 5.38 (s, 1H, H(7)), 8.35 (s, 1H, H(5)), 11.70 (br.s, 1H, NH) ppm.

Adduct with 2,4,6-trinitrotoluene: **6-Nitro-7-(2,4,6-trinitrobenzyl)-4,7-dihydro[1,2,5]oxadiazolo[3,4-*b*]pyridine 1-oxide** (**15**).

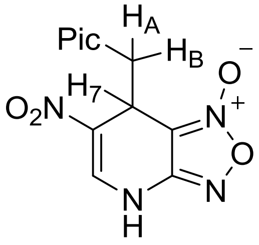

15

M.p. 122-123°C (dec.); ^{1}H NMR (DMSO-d_6, 300.13 MHz, δ): 3.30-3.38 (dd, J=14.1 Hz, J=8.2 Hz, 1H, CH_2), 3.74 (dd, J=14.1 Hz, J=7.2 Hz, 1H, CH_2), 4.97 (t, J=7.6 Hz, 1H, H(7)), 8.47 (s, 1H, H(5)), 9.10 (s, 2H, Pic), 11.85 (br.s., 1H, NH) ppm; ^{13}C NMR (DMSO-d_6, 75.47 MHz, δ): 30.33, 31.96, 107.05, 122.80, 125.55, 130.85, 138.79, 146.73, 151.19, 151.22 ppm; IR (KBr, ν_{max}/cm^{-1}): 722, 818, 986, 1022, 1084, 1205, 1251, 1321, 1349, 1491, 1544, 1587, 1653, 3093; HRMS (ESI): $[C_{12}H_7N_7O_{10} + NH_4]^+$ calc. 427.0593, found 427.0575. anal. calcd for $C_{12}H_7N_7O_{10}$ (%): C, 35.22; H, 1.72; N, 23.96; found C, 35.37; H, 1.58; N, 23.74.

NMR and HRMS spectra of compounds 6–15

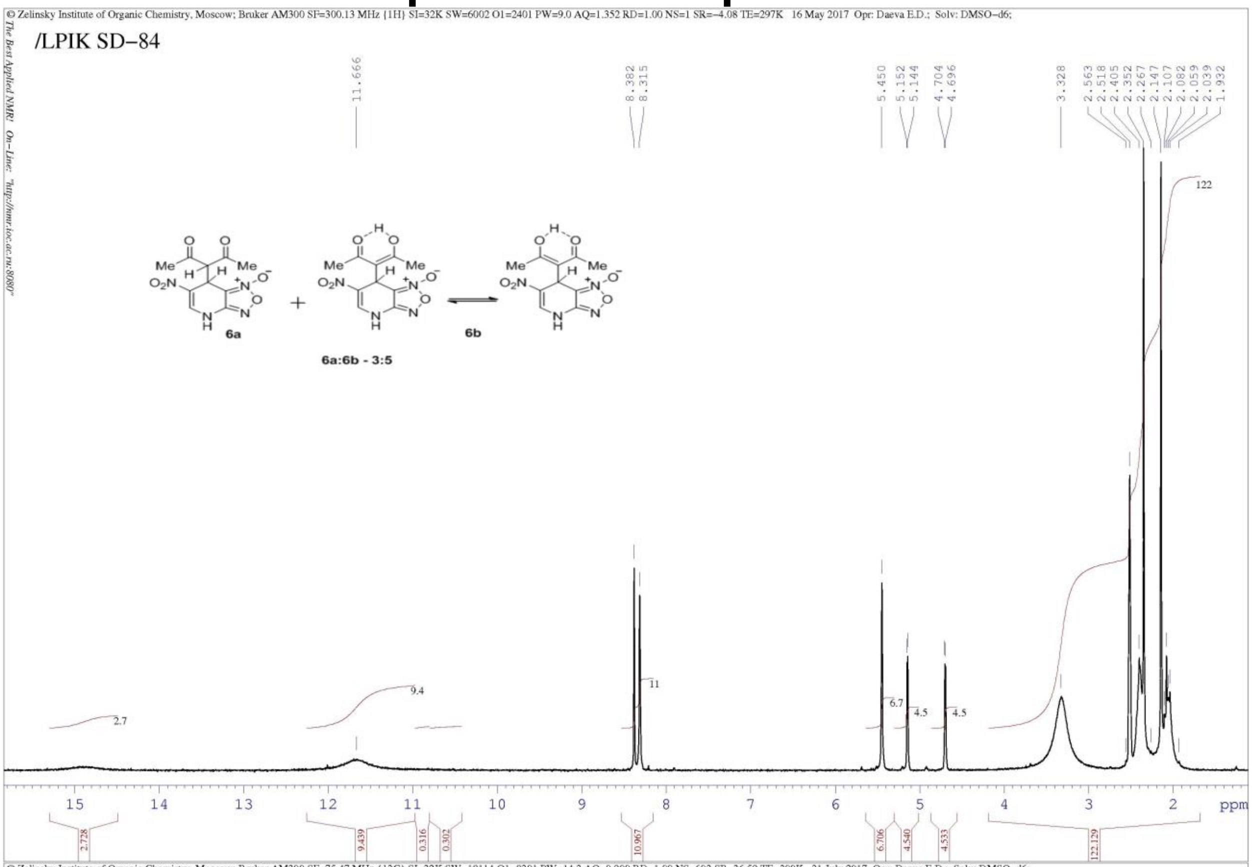

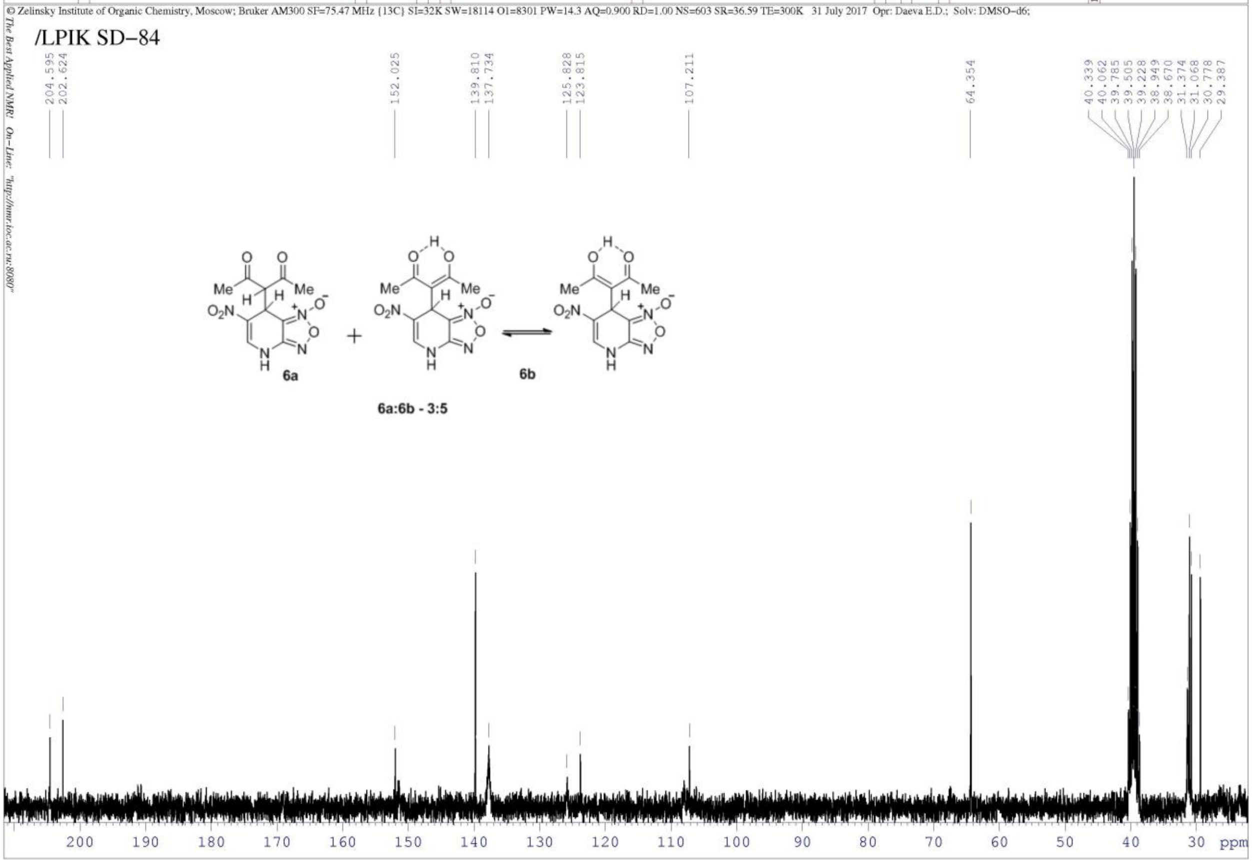

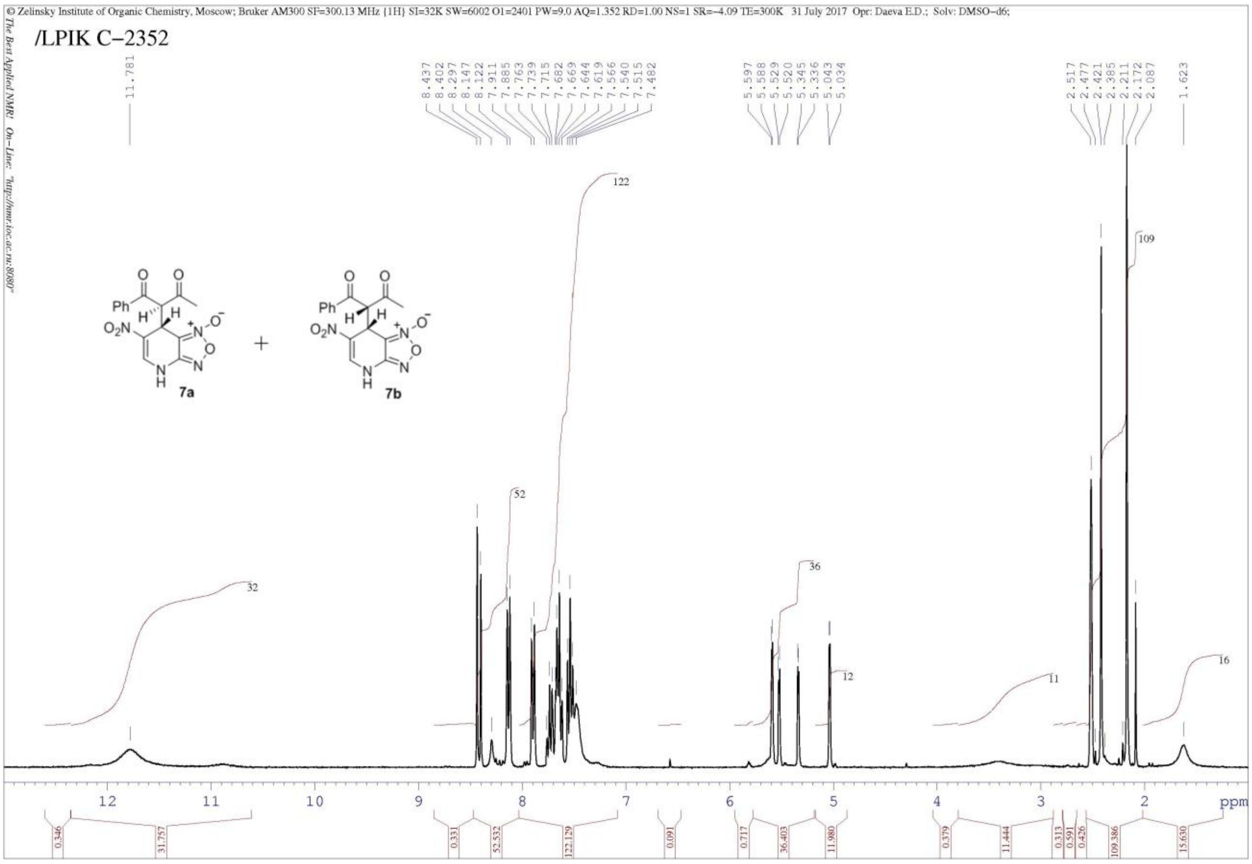
© Zelinsky Institute of Organic Chemistry, Moscow; Bruker AM300 SF=300.13 MHz {1H} SI=32K SW=6002 O1=2401 PW=9.0 AQ=1.352 RD=1.00 NS=1 SR=-4.09 TE=300K 31 July 2017 Opr: Daeva E.D.; Solv: DMSO-d6;
The Best Applied NMR! On-Line: "http://nmr.ioc.ac.ru:8080"
/LPIK C-2352
11.781
8.437 8.402 8.297 8.147 8.122 7.911 7.885 7.763 7.739 7.715 7.682 7.669 7.644 7.619 7.566 7.540 7.515 7.482
5.597 5.588 5.529 5.520 5.345 5.336 5.043 5.034
2.517 2.477 2.421 2.385 2.211 2.172 2.087
1.623
7a
7b
ppm
0.346 31.757 0.331 52.532 122.129 0.091 0.717 36.403 11.980 0.379 11.444 0.313 0.591 0.426 109.386 15.630

Display Report

Analysis Info

Analysis Name	D:\Data\Kolotyrkina\2017\Starosotnikov\0926012.d	Acquisition Date	26.09.2017 12:49:10
Method	tune_low.m	Operator	BDAL@DE
Sample Name	/LPIK C-2352	Instrument / Ser#	micrOTOF 10248
Comment	C15H12N4O6 mH 345.0829 calibrant added		

Acquisition Parameter

Source Type	ESI	Ion Polarity	Positive	Set Nebulizer	0.4 Bar
Focus	Not active			Set Dry Heater	180 °C
Scan Begin	50 m/z	Set Capillary	4500 V	Set Dry Gas	4.0 l/min
Scan End	3000 m/z	Set End Plate Offset	-500 V	Set Divert Valve	Waste

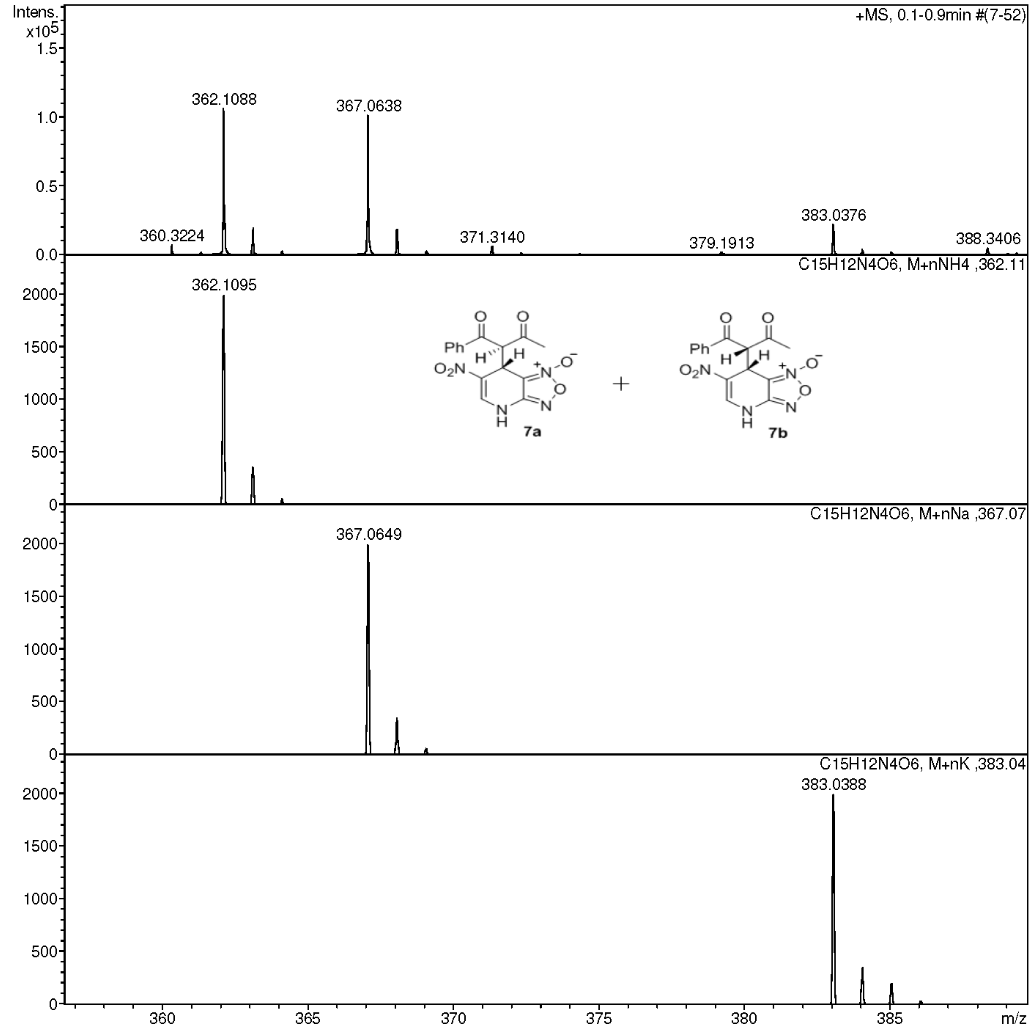

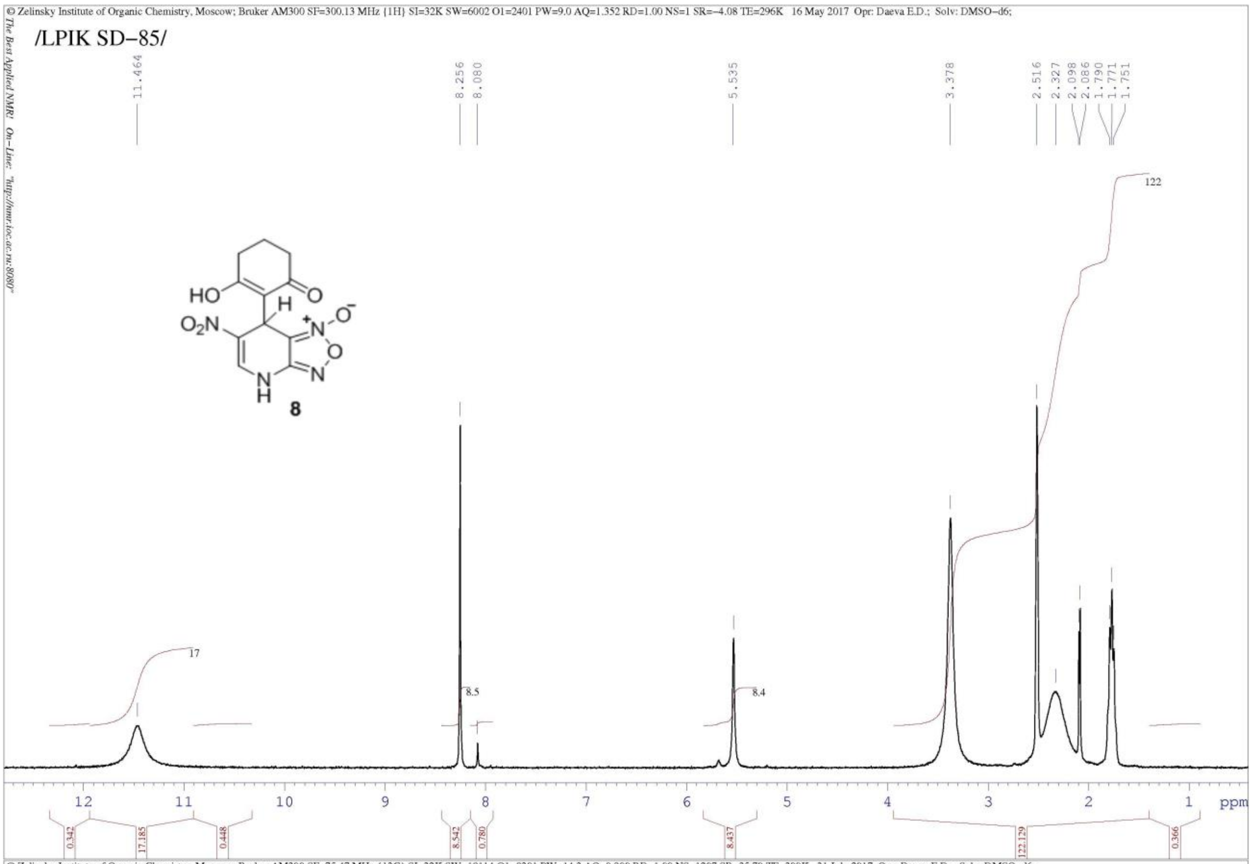
© Zelinsky Institute of Organic Chemistry, Moscow; Bruker AM300 SF=300.13 MHz {1H} SI=32K SW=6002 O1=2401 PW=9.0 AQ=1.352 RD=1.00 NS=1 SR=−4.08 TE=296K 16 May 2017 Opr: Daeva E.D.; Solv: DMSO−d6;
The Best Applied NMR! On−Line: "http://nmr.ioc.ac.ru:8080"
/LPIK SD−85/
11.464
8.256
8.080
5.535
3.378
2.516
2.327
2.098
2.086
1.790
1.771
1.751
HO
O
H
O_2N
N
O
N
O
N
H
8
122
17
8.5
8.4
12 11 10 9 8 7 6 5 4 3 2 1 ppm
0.342
17.185
0.448
8.542
0.780
8.437
122.129
0.366

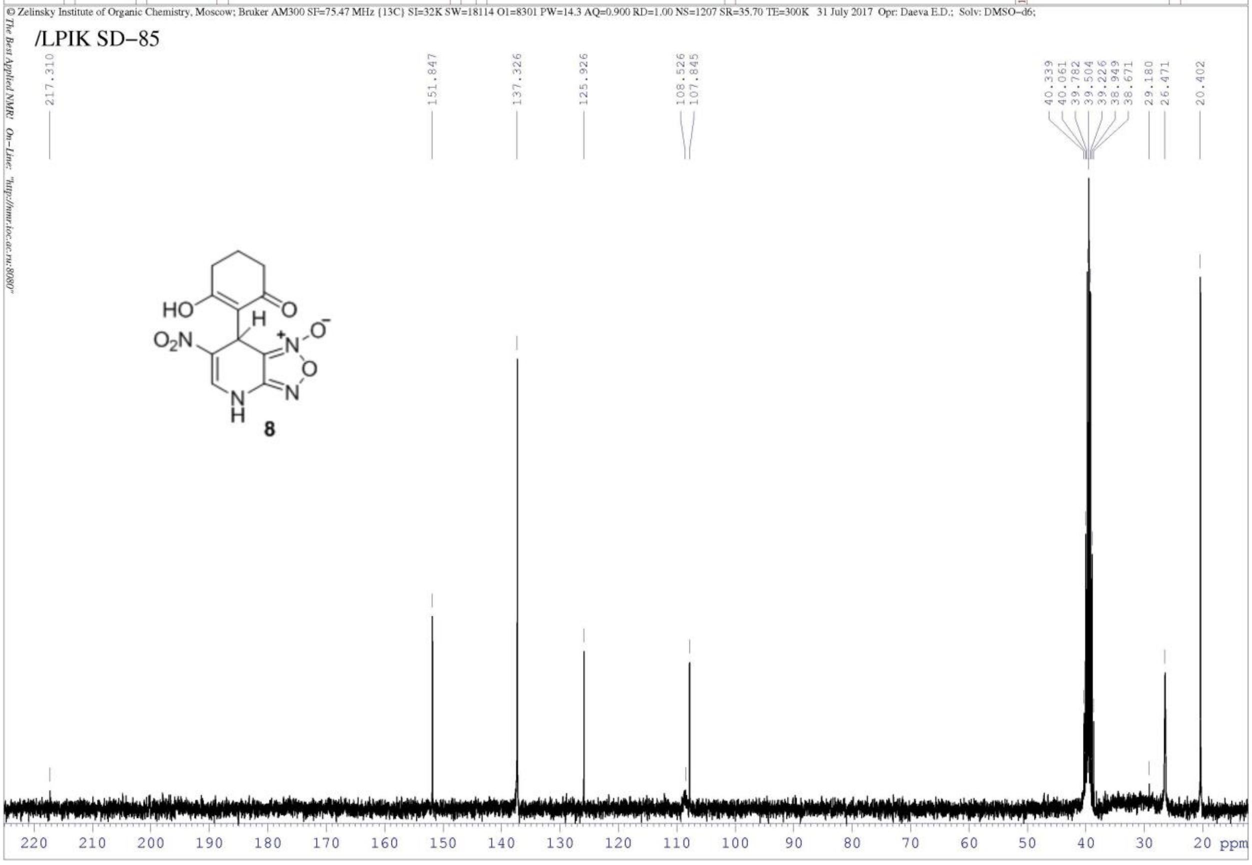
© Zelinsky Institute of Organic Chemistry, Moscow; Bruker AM300 SF=75.47 MHz {13C} SI=32K SW=18114 O1=8301 PW=14.3 AQ=0.900 RD=1.00 NS=1207 SR=35.70 TE=300K 31 July 2017 Opr: Daeva E.D.; Solv: DMSO−d6;
The Best Applied NMR! On−Line: "http://nmr.ioc.ac.ru:8080"
/LPIK SD−85
217.310
151.847
137.326
125.926
108.526
107.845
40.339
40.061
39.782
39.504
39.226
38.949
38.671
29.180
26.471
20.402
HO
O
H
O_2N
N
O
N
O
N
H
8
220 210 200 190 180 170 160 150 140 130 120 110 100 90 80 70 60 50 40 30 20 ppm

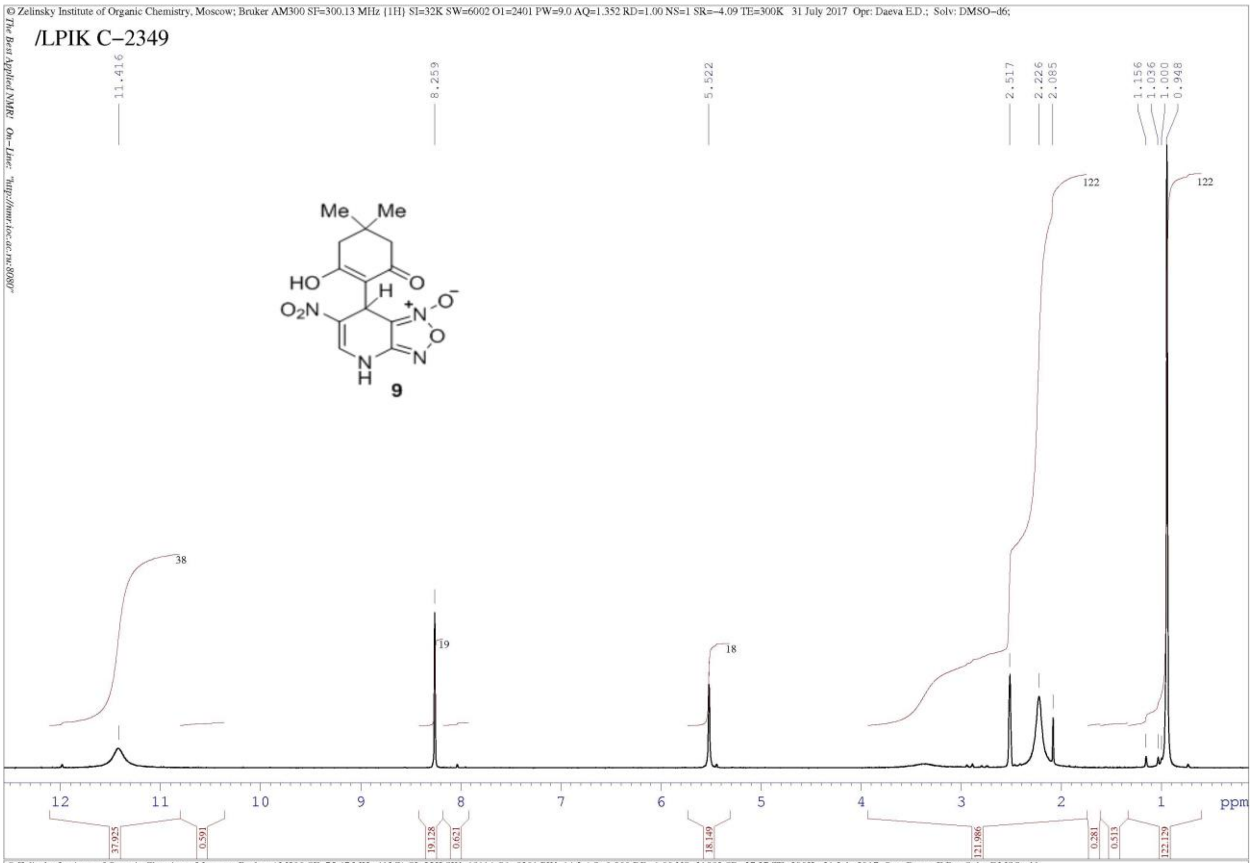
© Zelinsky Institute of Organic Chemistry, Moscow; Bruker AM300 SF=300.13 MHz {1H} SI=32K SW=6002 O1=2401 PW=9.0 AQ=1.352 RD=1.00 NS=1 SR=-4.09 TE=300K 31 July 2017 Opr: Daeva E.D.; Solv: DMSO-d6;
The Best Applied NMR! On-Line: "http://nmr.ioc.ac.ru:8080"
/LPIK C-2349
11.416
8.259
5.522
2.517
2.226
2.085
1.156
1.036
1.000
0.948
Me
Me
HO
O
H
O_2N
N
O
H
9
ppm
37.925
0.591
19.128
0.621
18.149
121.986
0.281
0.513
122.129

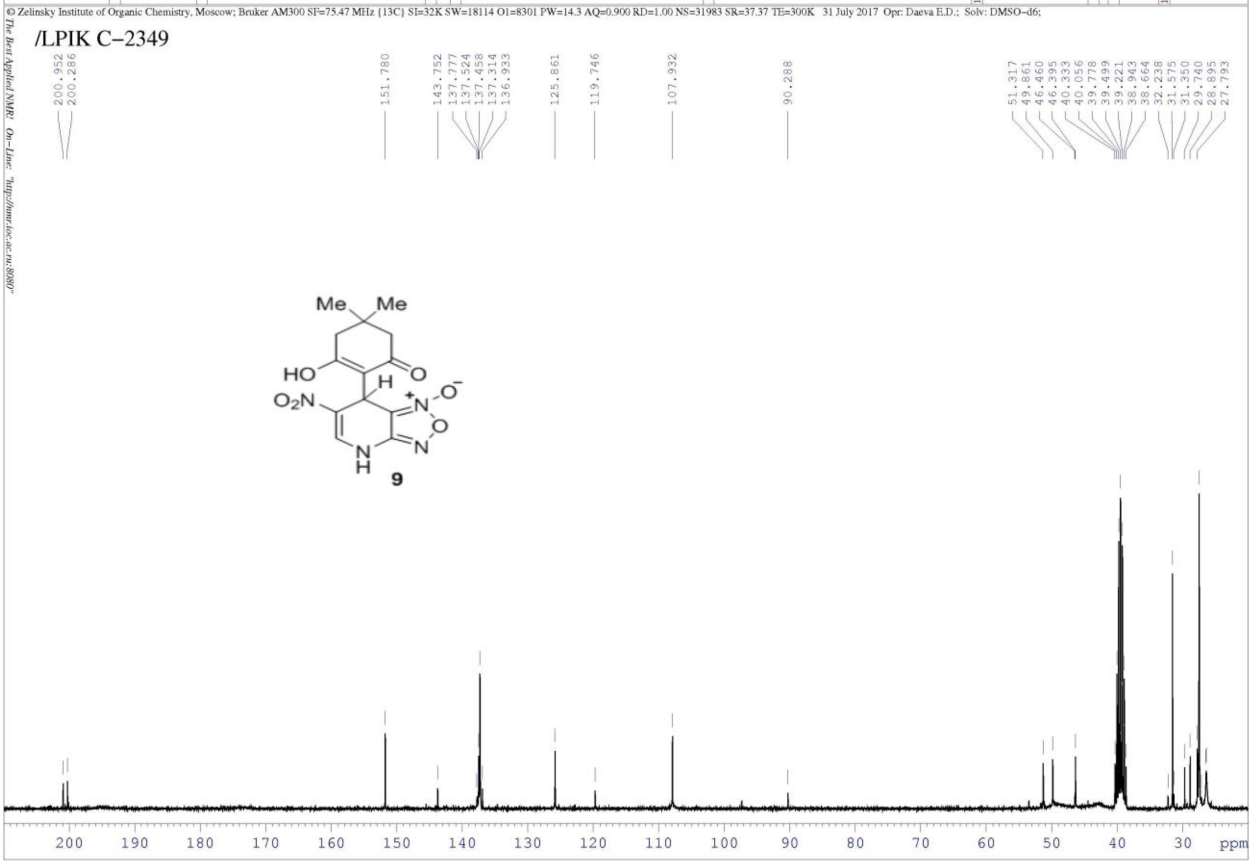
© Zelinsky Institute of Organic Chemistry, Moscow; Bruker AM300 SF=75.47 MHz {13C} SI=32K SW=18114 O1=8301 PW=14.3 AQ=0.900 RD=1.00 NS=31983 SR=37.37 TE=300K 31 July 2017 Opr: Daeva E.D.; Solv: DMSO-d6;
The Best Applied NMR! On-Line: "http://nmr.ioc.ac.ru:8080"
/LPIK C-2349
200.952
200.286
151.780
143.752
137.777
137.524
137.458
137.314
136.933
125.861
119.746
107.932
90.288
51.317
49.861
46.460
46.395
40.333
40.056
39.778
39.499
39.221
38.943
38.664
32.258
31.575
31.350
29.740
28.895
27.793
Me
Me
HO
O
H
O_2N
N
O
H
9
ppm

Display Report

Analysis Info

Analysis Name	D:\Data\Kolotyrkina\2017\Starosotnikov\0926011.d	Acquisition Date	26.09.2017 12:42:22
Method	tune_low.m	Operator	BDAL@DE
Sample Name	/LPIK C-2349	Instrument / Ser#	micrOTOF 10248
Comment	C13H14N4O6 mH 323.0986 calibrant added		

Acquisition Parameter

Source Type	ESI	Ion Polarity	Positive	Set Nebulizer	0.4 Bar
Focus	Not active			Set Dry Heater	180 °C
Scan Begin	50 m/z	Set Capillary	4500 V	Set Dry Gas	4.0 l/min
Scan End	3000 m/z	Set End Plate Offset	-500 V	Set Divert Valve	Waste

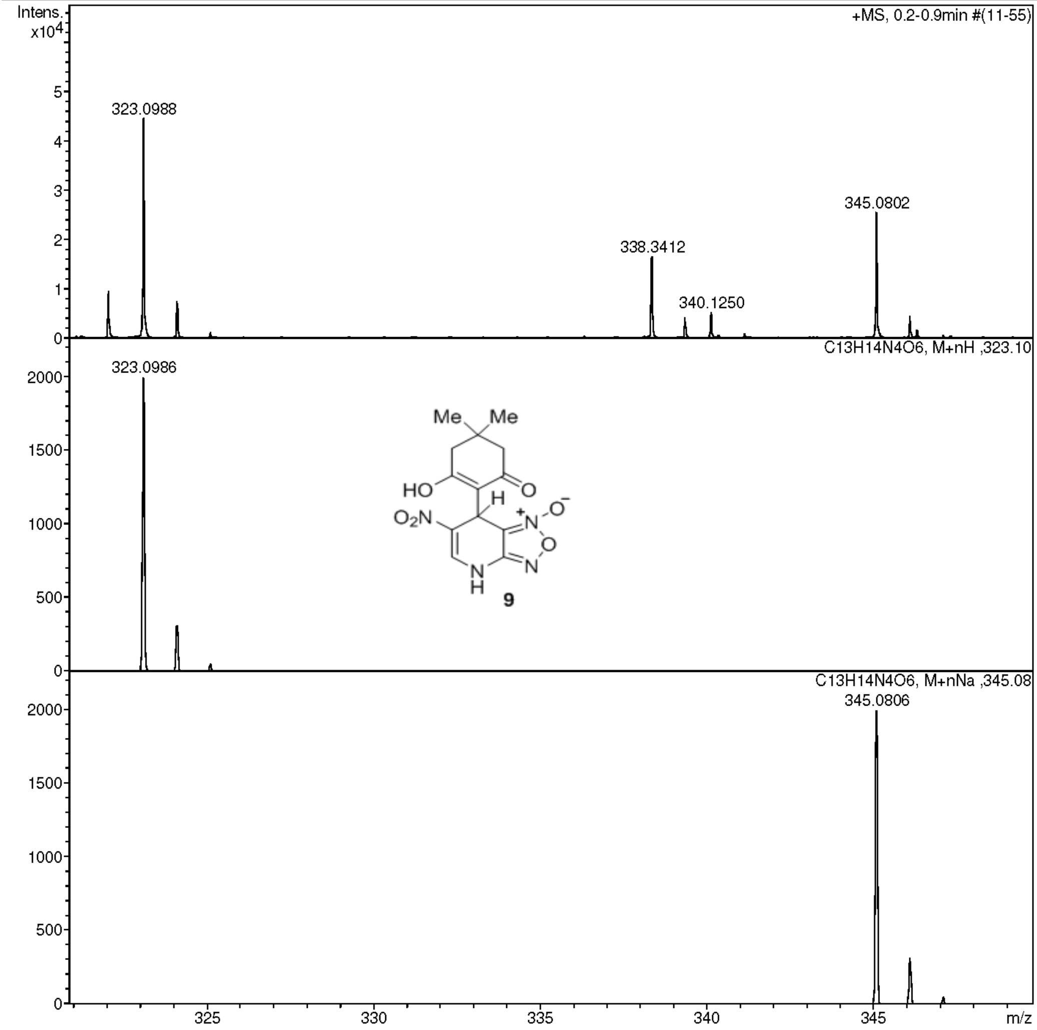

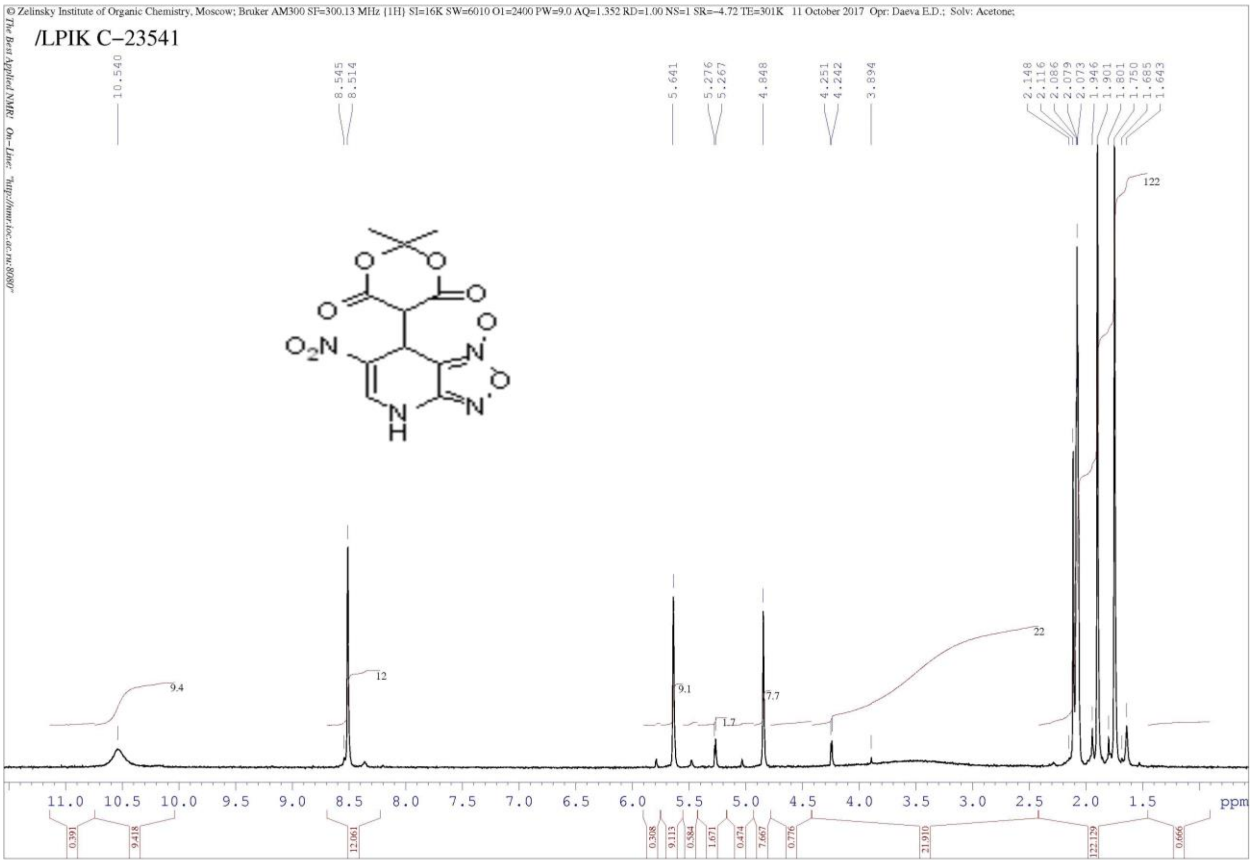
© Zelinsky Institute of Organic Chemistry, Moscow; Bruker AM300 SF=300.13 MHz {1H} SI=16K SW=6010 O1=2400 PW=9.0 AQ=1.352 RD=1.00 NS=1 SR=-4.72 TE=301K 11 October 2017 Opr: Daeva E.D.; Solv: Acetone;
/LPIK C-23541
The Best Applied NMR! On-Line: "http://nmr.ioc.ac.ru:8080"
10.540
8.545
8.514
5.641
5.276
5.267
4.848
4.251
4.242
3.894
2.148
2.116
2.086
2.079
2.073
1.946
1.901
1.801
1.750
1.685
1.643
ppm

Display Report

Analysis Info

Analysis Name	D:\Data\Kolotyrkina\2017\Starosotnikov\0926013.d	Acquisition Date	26.09.2017 12:55:18
Method	tune_low.m	Operator	BDAL@DE
Sample Name	/LPIK C-2354	Instrument / Ser#	micrOTOF 10248
Comment	C11H10N4O8 mH 327.0571 calibrant added		

Acquisition Parameter

Source Type	ESI	Ion Polarity	Positive	Set Nebulizer	0.4 Bar
Focus	Not active			Set Dry Heater	180 °C
Scan Begin	50 m/z	Set Capillary	4500 V	Set Dry Gas	4.0 l/min
Scan End	3000 m/z	Set End Plate Offset	-500 V	Set Divert Valve	Waste

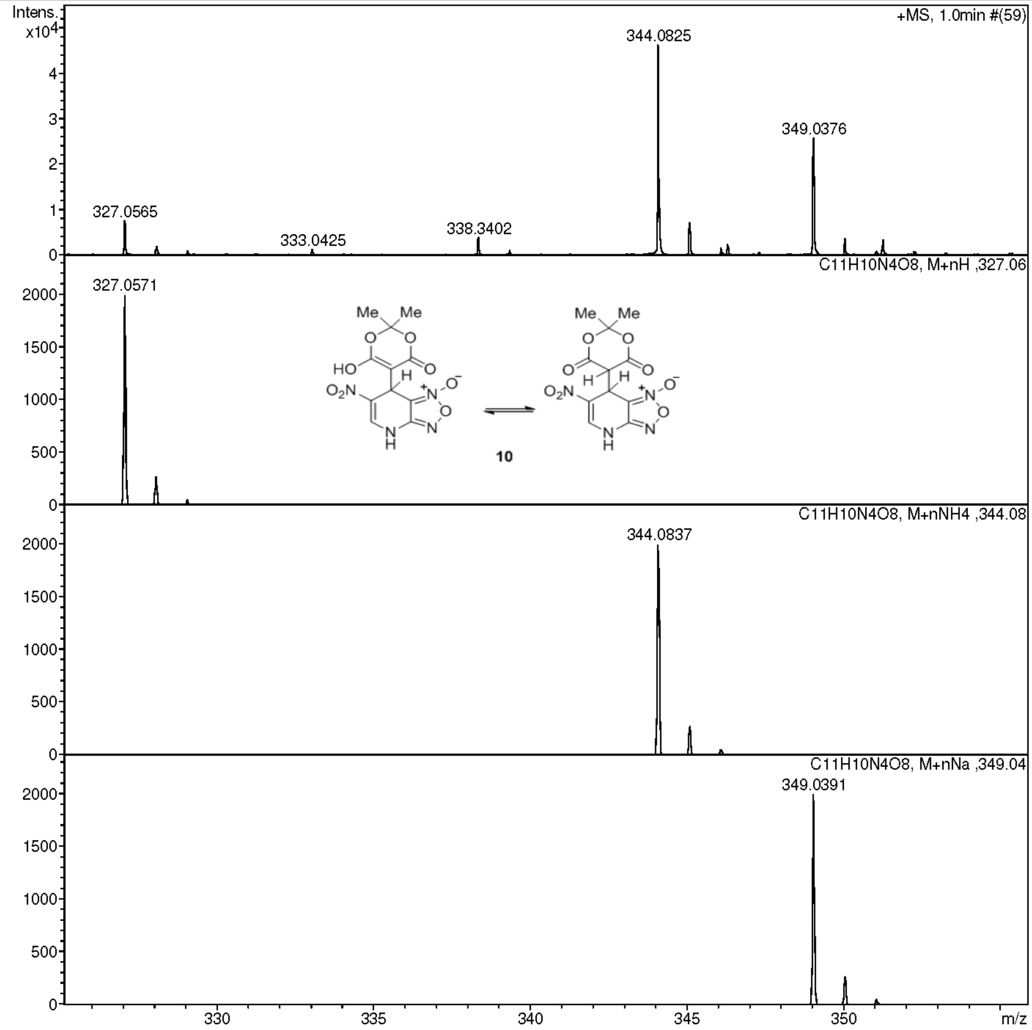

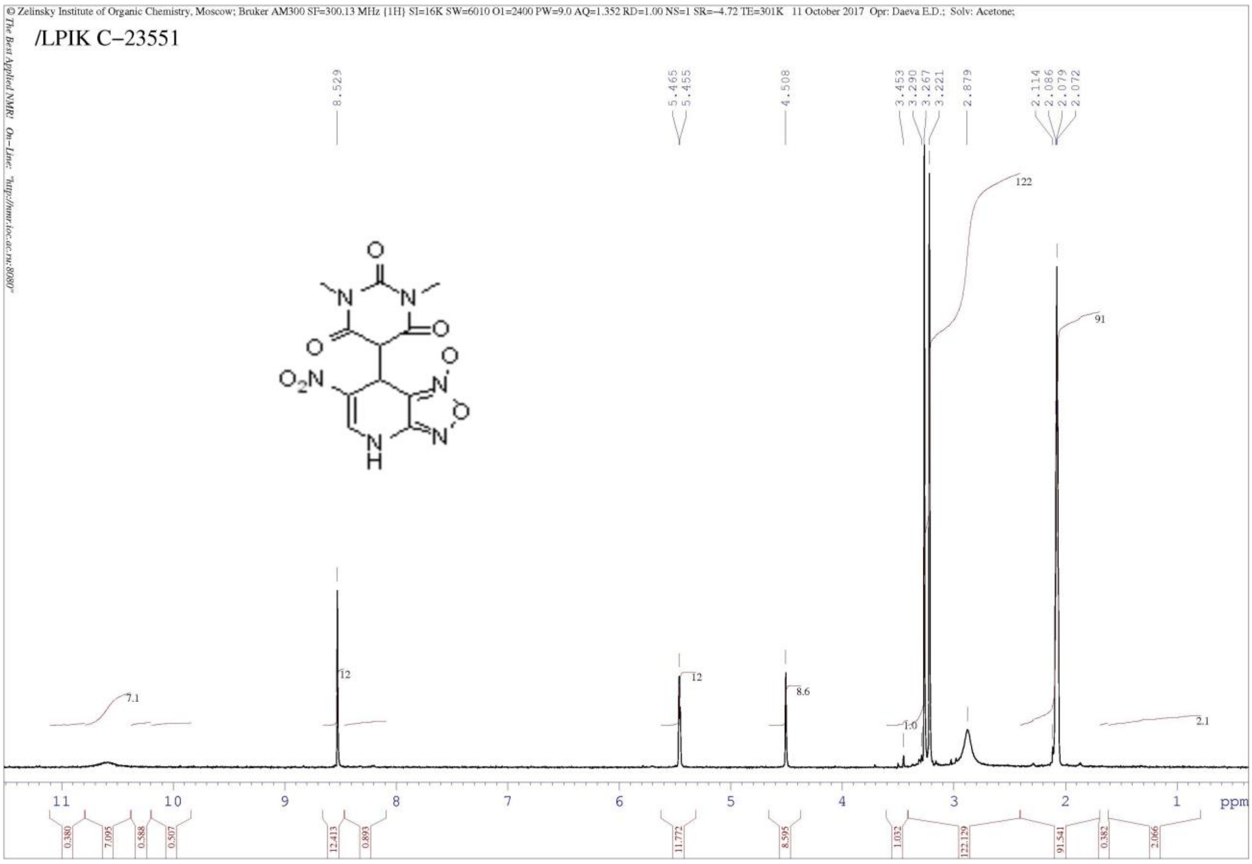
© Zelinsky Institute of Organic Chemistry, Moscow; Bruker AM300 SF=300.13 MHz {1H} SI=16K SW=6010 O1=2400 PW=9.0 AQ=1.352 RD=1.00 NS=1 SR=-4.72 TE=301K 11 October 2017 Opr: Daeva E.D.; Solv: Acetone;
The Best Applied NMR! On-Line: "http://nmr.ioc.ac.ru:8080"
/LPIK C-23551
8.529
5.465
5.455
4.508
3.453
3.290
3.267
3.221
2.879
2.114
2.086
2.079
2.072
122
91
7.1
12
12
8.6
1.0
2.1
11
10
9
8
7
6
5
4
3
2
1
ppm
0.380
7.095
0.588
0.507
12.413
0.893
11.772
8.595
1.032
122.129
91.541
0.382
2.066

Display Report

Analysis Info

Analysis Name	D:\Data\Kolotyrkina\2017\Starosotnikov\0926014.d	Acquisition Date	26.09.2017 13:06:38
Method	tune_low.m	Operator	BDAL@DE
Sample Name	/LPIK C-2355	Instrument / Ser#	micrOTOF 10248
Comment	C11H10N6O7 mH 339.0683 calibrant added		

Acquisition Parameter

Source Type	ESI	Ion Polarity	Positive	Set Nebulizer	0.4 Bar
Focus	Not active			Set Dry Heater	180 °C
Scan Begin	50 m/z	Set Capillary	4500 V	Set Dry Gas	4.0 l/min
Scan End	3000 m/z	Set End Plate Offset	-500 V	Set Divert Valve	Waste

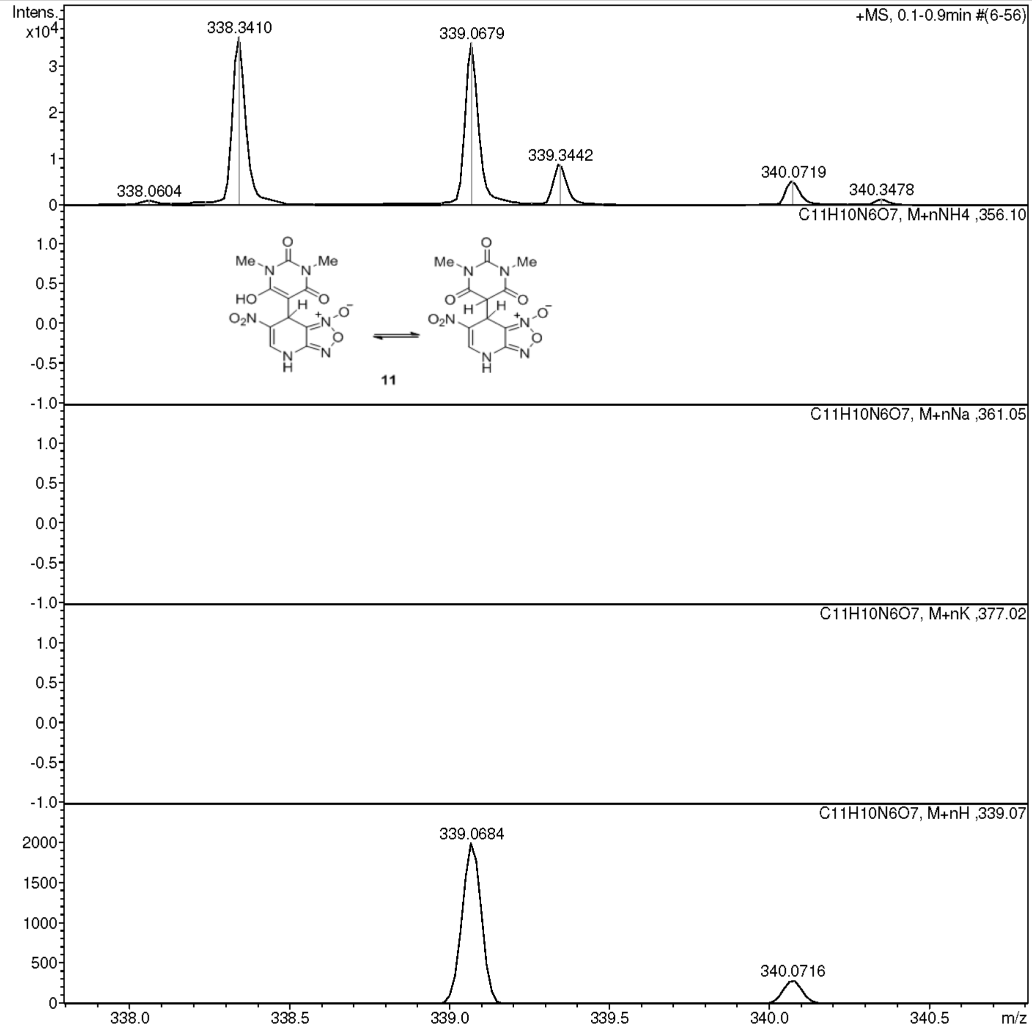

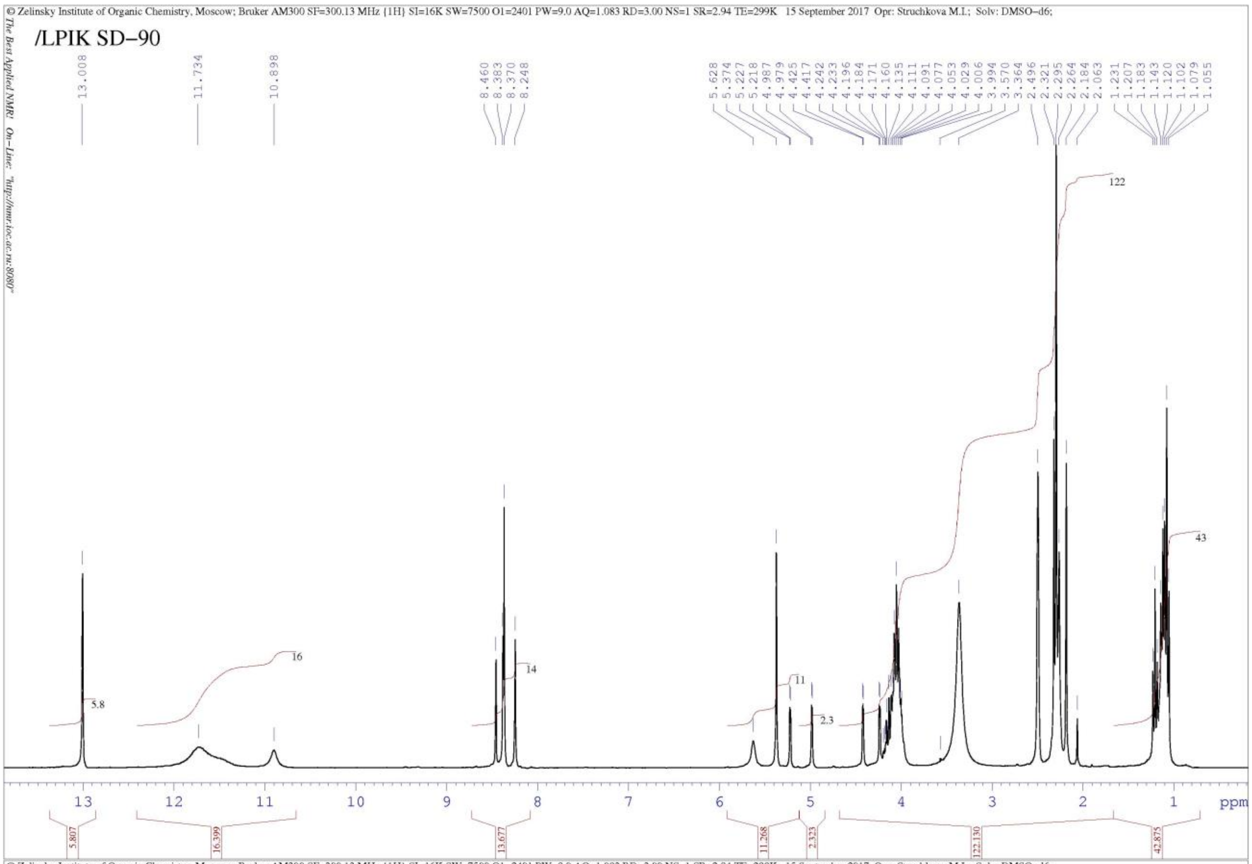
© Zelinsky Institute of Organic Chemistry, Moscow; Bruker AM300 SF=300.13 MHz {1H} SI=16K SW=7500 O1=2401 PW=9.0 AQ=1.083 RD=3.00 NS=1 SR=2.94 TE=299K 15 September 2017 Opr: Struchkova M.I.; Solv: DMSO–d6;
The Best Applied NMR! On–Line: "http://nmr.ioc.ac.ru:8080"
/LPIK SD–90
ppm

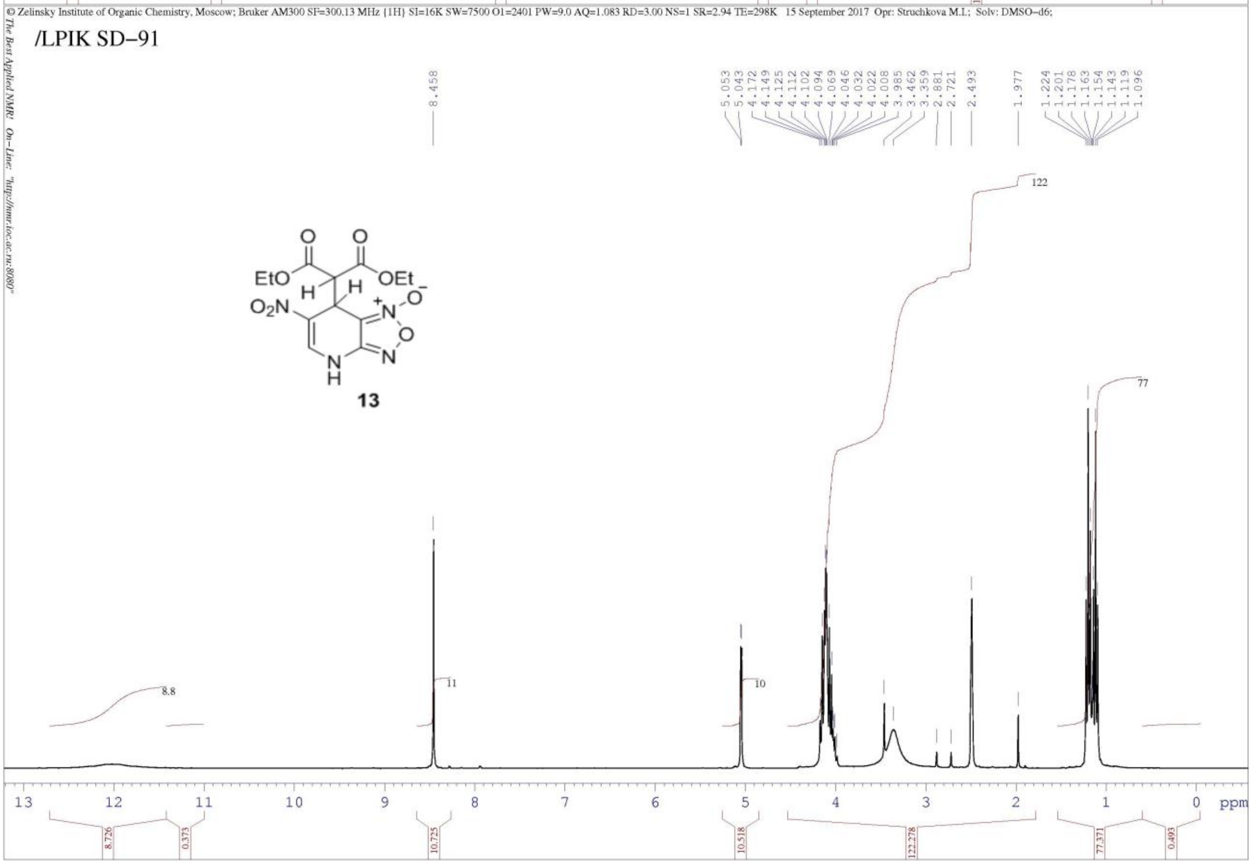
© Zelinsky Institute of Organic Chemistry, Moscow; Bruker AM300 SF=300.13 MHz {1H} SI=16K SW=7500 O1=2401 PW=9.0 AQ=1.083 RD=3.00 NS=1 SR=2.94 TE=298K 15 September 2017 Opr: Struchkova M.I.; Solv: DMSO–d6;
The Best Applied NMR! On–Line: "http://nmr.ioc.ac.ru:8080"
/LPIK SD–91
13
ppm

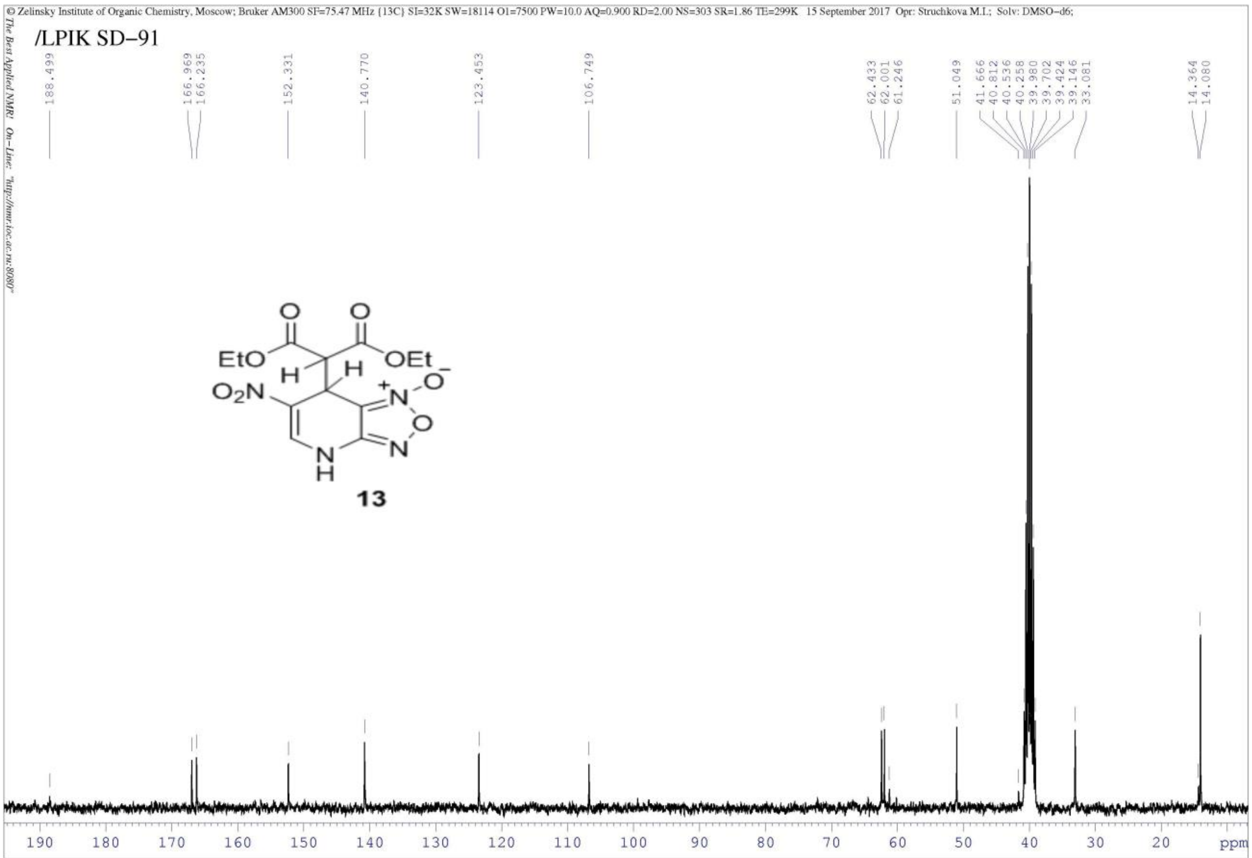
© Zelinsky Institute of Organic Chemistry, Moscow; Bruker AM300 SF=75.47 MHz {13C} SI=32K SW=18114 O1=7500 PW=10.0 AQ=0.900 RD=2.00 NS=303 SR=1.86 TE=299K 15 September 2017 Opr: Struchkova M.I.; Solv: DMSO−d6;
The Best Applied NMR! On−Line: "http://nmr.ioc.ac.ru:8080"
/LPIK SD−91
188.499
166.969
166.235
152.331
140.770
123.453
106.749
62.433
62.001
61.246
51.049
41.666
40.812
40.536
40.258
39.980
39.702
39.424
39.146
33.081
14.364
14.080
EtO
OEt
O2N
H
N
O
N
H
13
190 180 170 160 150 140 130 120 110 100 90 80 70 60 50 40 30 20 ppm

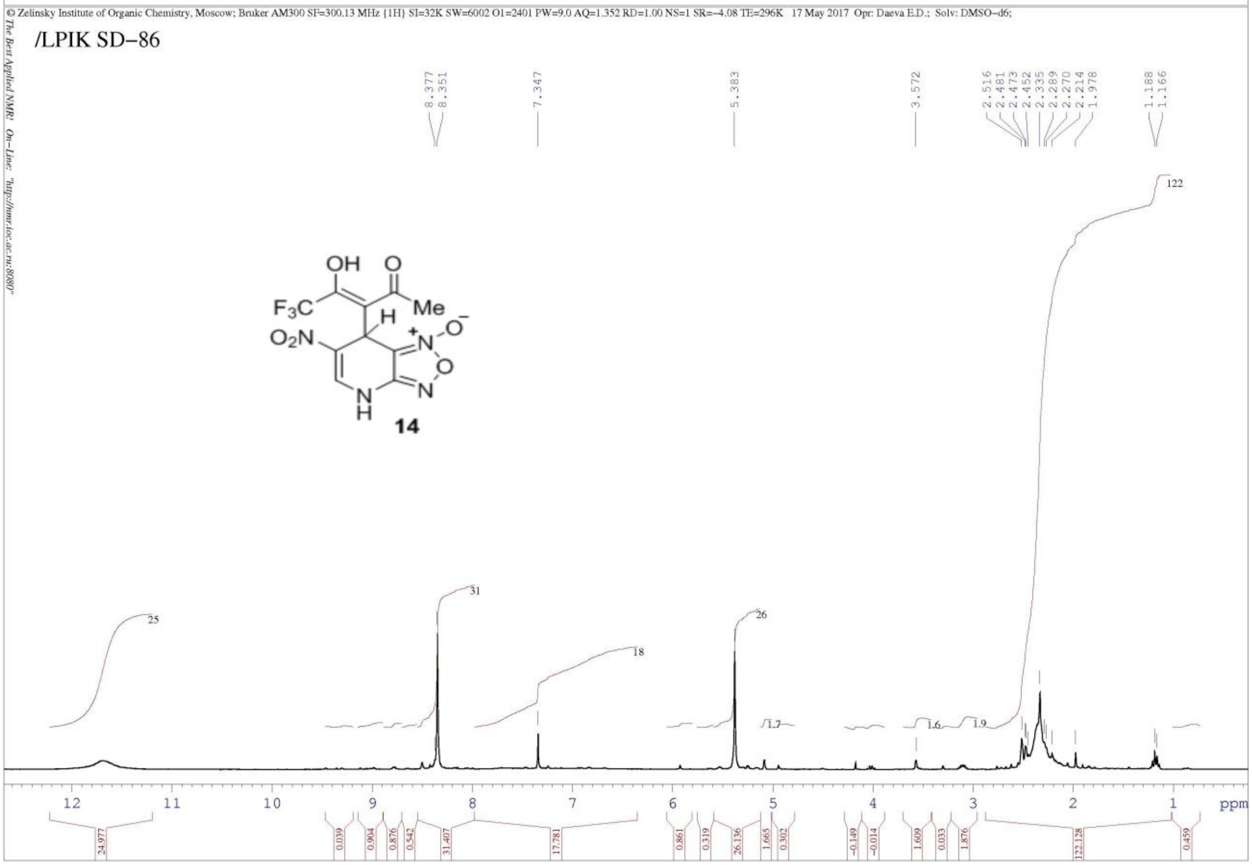
© Zelinsky Institute of Organic Chemistry, Moscow; Bruker AM300 SF=300.13 MHz {1H} SI=32K SW=6002 O1=2401 PW=9.0 AQ=1.352 RD=1.00 NS=1 SR=−4.08 TE=296K 17 May 2017 Opr: Daeva E.D.; Solv: DMSO−d6;
The Best Applied NMR! On−Line: "http://nmr.ioc.ac.ru:8080"
/LPIK SD−86
8.377
8.351
7.347
5.383
3.572
2.516
2.481
2.473
2.452
2.335
2.289
2.270
2.214
1.978
1.188
1.166
OH
F3C
Me
O2N
H
N
O
N
H
14
25
31
18
26
1.7
1.6
1.9
122
12 11 10 9 8 7 6 5 4 3 2 1 ppm
24.977
0.039
0.904
0.876
0.542
31.407
17.781
0.861
0.319
26.136
1.665
0.302
−0.149
−0.014
1.609
0.033
1.876
122.128
0.459

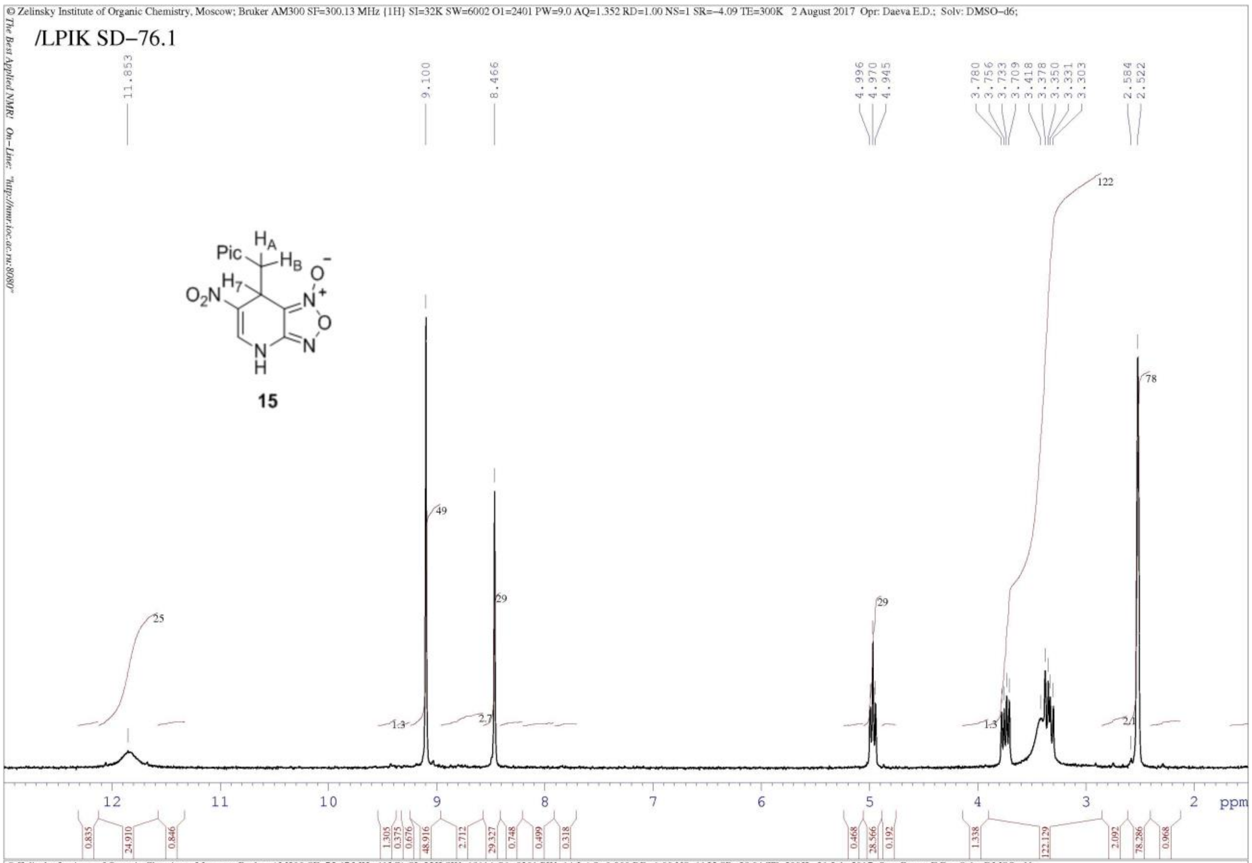
© Zelinsky Institute of Organic Chemistry, Moscow; Bruker AM300 SF=300.13 MHz {1H} SI=32K SW=6002 O1=2401 PW=9.0 AQ=1.352 RD=1.00 NS=1 SR=−4.09 TE=300K 2 August 2017 Opr: Daeva E.D.; Solv: DMSO−d6;
/LPIK SD−76.1
11.853
9.100
8.466
4.996
4.970
4.945
3.780
3.756
3.733
3.709
3.418
3.378
3.350
3.331
3.303
2.584
2.522
Pic
H_A
H_B
H_7
O_2N
N
O
N
H
15
ppm

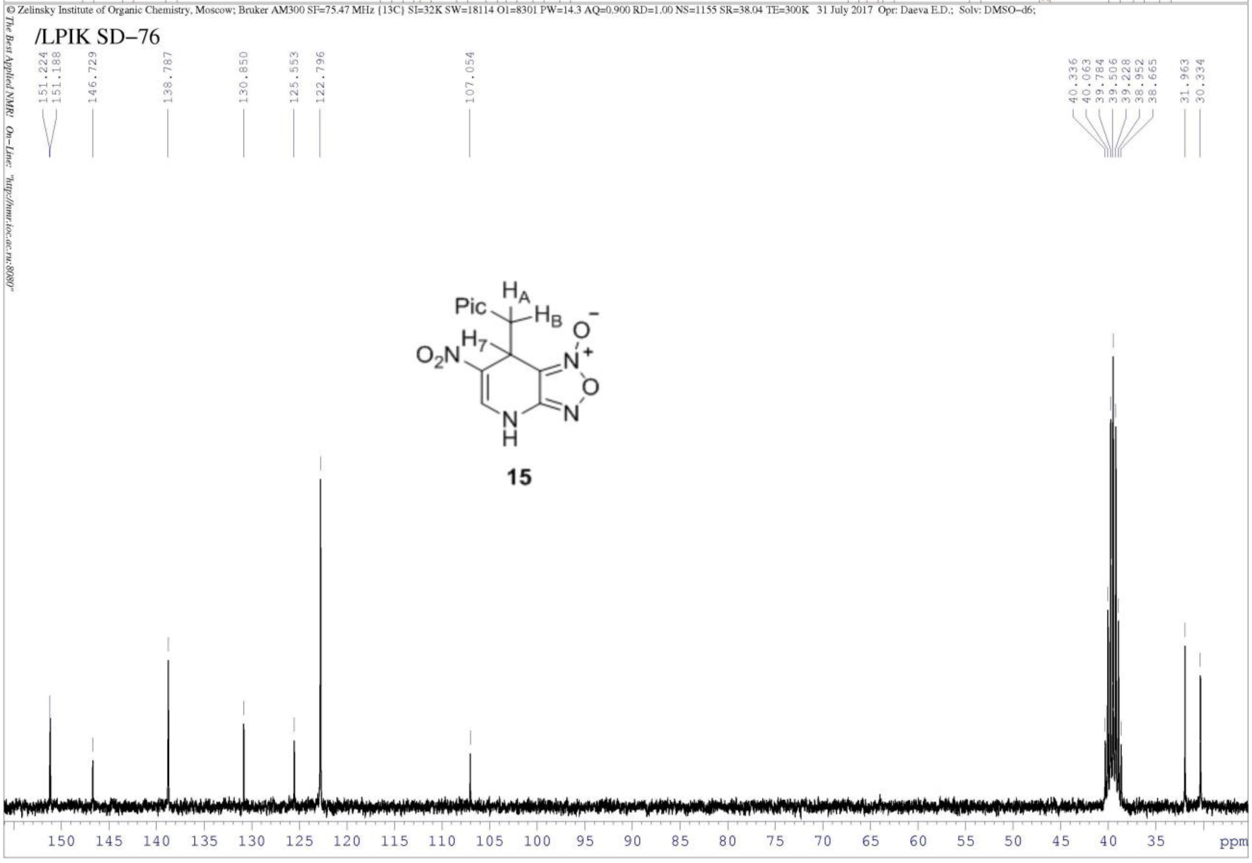
© Zelinsky Institute of Organic Chemistry, Moscow; Bruker AM300 SF=75.47 MHz {13C} SI=32K SW=18114 O1=8301 PW=14.3 AQ=0.900 RD=1.00 NS=1155 SR=38.04 TE=300K 31 July 2017 Opr: Daeva E.D.; Solv: DMSO−d6;
/LPIK SD−76
151.224
151.188
146.729
138.787
130.850
125.553
122.796
107.054
40.336
40.063
39.784
39.506
39.228
38.952
38.665
31.963
30.334
Pic
H_A
H_B
H_7
O_2N
N
O
N
H
15
ppm

Display Report

Analysis Info

Analysis Name	D:\Data\Kolotyrkina\2017\Starosotnikov\0926010.d	Acquisition Date	26.09.2017 12:32:11
Method	tune_low.m	Operator	BDAL@DE
Sample Name	/LPIK SD-81	Instrument / Ser#	micrOTOF 10248
Comment	C12H7N7O10 mH 410.0327 calibrant added		

Acquisition Parameter

Source Type	ESI	Ion Polarity	Positive	Set Nebulizer	0.4 Bar
Focus	Not active			Set Dry Heater	180 °C
Scan Begin	50 m/z	Set Capillary	4500 V	Set Dry Gas	4.0 l/min
Scan End	3000 m/z	Set End Plate Offset	-500 V	Set Divert Valve	Waste

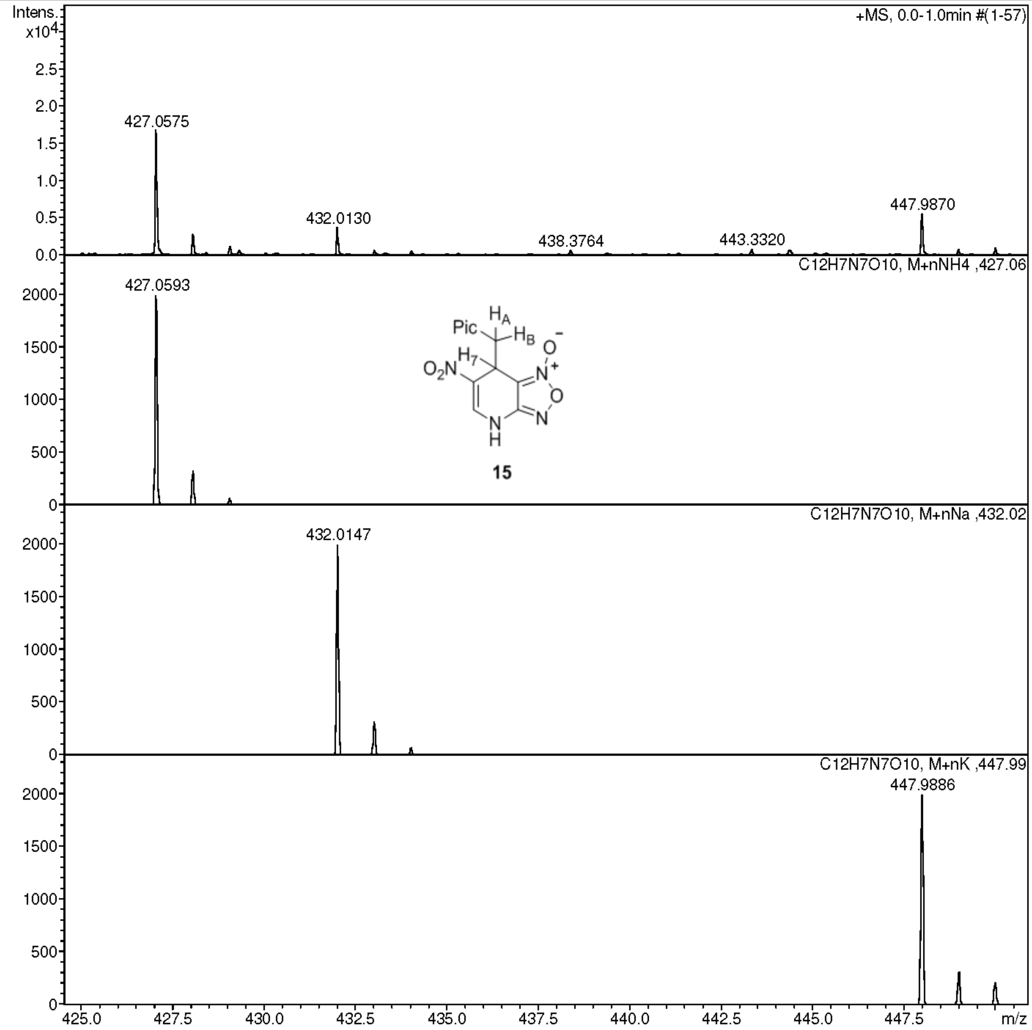

X-ray diffraction experiments:

Data collection was performed on a Bruker APEX DUO diffractometer equipped with Apex II CCD detector and operating with Mo Kα radiation (λ = 0.71073 Å). Frames were integrated using the Bruker SAINT software package [SAINT (version 8.34A), Bruker AXS Inc., Madison, Wisconsin, USA, 2014] by a narrow-frame algorithm. A semiempirical absorption correction was applied with the SADABS program [G.M. Sheldrick, SADABS, v. 2008/1, Bruker/Siemens Area Detector Absorption Correction Program, Bruker AXS, Madison, Wisconsin, USA, 2008] using the intensity data of equivalent reflections. The structures were solved with direct methods and refined by the full-matrix least-squares technique against F^2_{hkl} in anisotropic approximation with SHELX [G.M. Sheldrick, Acta Cryst. 2015, C71, 3-8] software package. Hydrogen atoms connected to oxygen and nitrogen atoms were located from difference Fourier synthesis and refined isotropically. All other hydrogen atoms were placed in calculated positions and refined in riding model U_{iso}(H) equal to 1.5U_{eq}(Cm) and 1.2U_{eq}(Ci), where U_{eq}(Cm) and 1.2U_{eq}(Ci) are respectively the equivalent thermal parameters of methyl and all other carbon atoms to which corresponding H atoms are bonded. Detailed crystallographic information is given in Tables S1 and S2. Crystallographic data have been deposited to the Cambridge Crystallographic Data Centre, CCDC numbers 1574256 **(12·DMF)** and 1574257 **(15·DMSO),** and can be retrieved free of charge via https://www.ccdc.cam.ac.uk/structures.

Table S1. Crystallographic data for **12·DMF** and **15·DMSO**.

	**(12·DMF)**	**(15·DMSO)**
Formula	$C_{14}H_{19}N_5O_8$	$C_{14}H_{13}N_7O_{11}S$
Formula weight	385.34	487.37
T, K	120	120
Crystal system	monoclinic	monoclinic
Space group	$P2_1/c$	$P2_1/n$
Z / Z'	4 / 1	4 / 1
a, Å	11.138(2)	10.9841(16)
b, Å	12.765(3)	7.2477(11)
c, Å	12.649(2)	24.162(3)
β, °	103.045(4)	99.107(3)
V, Å^3	1752.1(6)	1899.3(5)
d_{calc}, g cm^{-3}	1.461	1.704
μ, cm^{-1}	1.21	2.52
$2\theta_{max}$, °	60.0	60.0
Reflns. collected / independent	22965 / 5112	32671 / 5550
Observed reflections [I>2σ(I)]	3840	4079
R_1	0.0425	0.0463
wR_2	0.1153	0.0986
GOF	1.032	1.045
Residual density, e Å^{-3} (d_{max}/d_{min})	0.336/-0.271	0.386/-0.350

Table S2. Selected bond lengths (Å) in crystals **12·DMF** and **15·DMSO**.

	**(12·DMF)**	**(15·DMSO)**
N1-C2	1.3610(18)	1.360(2)
C2-C3	1.3486(19)	1.350(2)
C3-C4	1.5175(17)	1.512(2)
C4-C5	1.4972(18)	1.489(2)
N7-C5	1.3104(16)	1.312(2)
O8-N7	1.4482(15)	1.448(2)
N7-O10	1.2301(14)	1.228(2)
O8-N9	1.3994(15)	1.393(2)
N9-C6	1.3068(17)	1.310(2)
N1-C6	1.3710(17)	1.366(2)
C5-C6	1.4110(18)	1.402(2)
C4-C11	1.5250(18)	1.567(2)
C11-C12	1.3687(19)	
C11-C14	1.450(2)	
O1-C14	1.2352(17)	
O2-C14	1.3344(17)	
O3-C12	1.3386(18)	
C11-C12		1.507(2)
C12-C13		1.389(2)
C12-C17		1.395(2)

checkCIF/PLATON report

Structure factors have been supplied for datablock(s) sd76

THIS REPORT IS FOR GUIDANCE ONLY. IF USED AS PART OF A REVIEW PROCEDURE FOR PUBLICATION, IT SHOULD NOT REPLACE THE EXPERTISE OF AN EXPERIENCED CRYSTALLOGRAPHIC REFEREE.

No syntax errors found.   CIF dictionary   Interpreting this report

Datablock: sd76

```
Bond precision:   C-C = 0.0022 A          Wavelength=0.71073

Cell:             a=10.9841(16)     b=7.2477(11)       c=24.162(3)
                  alpha=90          beta=99.107(3)     gamma=90
Temperature:      120 K

                 Calculated                  Reported
Volume           1899.3(5)                   1899.3(5)
Space group      P 21/n                      P 21/n
Hall group       -P 2yn                      -P 2yn
Moiety formula   C12 H7 N7 O10, C2 H6 O S    C12 H7 N7 O10, C2 H6 O S
Sum formula      C14 H13 N7 O11 S            C14 H13 N7 O11 S
Mr               487.37                      487.37
Dx,g cm-3        1.704                       1.704
Z                4                           4
Mu (mm-1)        0.252                       0.252
F000             1000.0                      1000.0
F000'            1001.10
h,k,lmax         15,10,33                    15,10,33
Nref             5550                        5550
Tmin,Tmax        0.964,0.980                 0.827,0.862
Tmin'            0.923

Correction method= # Reported T Limits: Tmin=0.827 Tmax=0.862
AbsCorr = MULTI-SCAN

Data completeness= 1.000          Theta(max)= 29.996

R(reflections)= 0.0463( 4079)     wR2(reflections)= 0.0986( 5550)

S = 1.045                 Npar= 323
```

The following ALERTS were generated. Each ALERT has the format
test-name_ALERT_alert-type_alert-level.
Click on the hyperlinks for more details of the test.

Alert level C

```
PLAT230_ALERT_2_C Hirshfeld Test Diff for    O4     --  N2      ..        6.3 s.u.
PLAT906_ALERT_3_C Large K value in the Analysis of Variance ......      2.658 Check
```

Alert level G

```
PLAT301_ALERT_3_G Main Residue  Disorder ..............(Resd  1)..         7% Note
PLAT395_ALERT_2_G Deviating  X-O-Y  Angle from 120 Deg for  O8          107.9 Degree
PLAT432_ALERT_2_G Short Inter X...Y Contact  O20"   ..  C14     ..       2.91 Ang.
PLAT720_ALERT_4_G Number of Unusual/Non-Standard Labels ..........          6 Note
PLAT793_ALERT_4_G The Model has Chirality at C4      (Centro SPGR)          R Verify
PLAT960_ALERT_3_G Number of Intensities with I  <   - 2*sig(I) ...          8 Check
PLAT978_ALERT_2_G Number C-C Bonds with Positive Residual Density.         13 Info
```

```
   0 ALERT level A = Most likely a serious problem - resolve or explain
   0 ALERT level B = A potentially serious problem, consider carefully
   2 ALERT level C = Check. Ensure it is not caused by an omission or oversight
   7 ALERT level G = General information/check it is not something unexpected

   0 ALERT type 1 CIF construction/syntax error, inconsistent or missing data
   4 ALERT type 2 Indicator that the structure model may be wrong or deficient
   3 ALERT type 3 Indicator that the structure quality may be low
   2 ALERT type 4 Improvement, methodology, query or suggestion
   0 ALERT type 5 Informative message, check
```

It is advisable to attempt to resolve as many as possible of the alerts in all categories. Often the minor alerts point to easily fixed oversights, errors and omissions in your CIF or refinement strategy, so attention to these fine details can be worthwhile. In order to resolve some of the more serious problems it may be necessary to carry out additional measurements or structure refinements. However, the purpose of your study may justify the reported deviations and the more serious of these should normally be commented upon in the discussion or experimental section of a paper or in the "special_details" fields of the CIF. checkCIF was carefully designed to identify outliers and unusual parameters, but every test has its limitations and alerts that are not important in a particular case may appear. Conversely, the absence of alerts does not guarantee there are no aspects of the results needing attention. It is up to the individual to critically assess their own results and, if necessary, seek expert advice.

Publication of your CIF in IUCr journals

A basic structural check has been run on your CIF. These basic checks will be run on all CIFs submitted for publication in IUCr journals (*Acta Crystallographica, Journal of Applied Crystallography, Journal of Synchrotron Radiation*); however, if you intend to submit to *Acta Crystallographica Section C* or *E* or *IUCrData*, you should make sure that full publication checks are run on the final version of your CIF prior to submission.

Publication of your CIF in other journals

Please refer to the *Notes for Authors* of the relevant journal for any special instructions relating to CIF submission.

PLATON version of 13/08/2017; check.def file version of 27/07/2017

Datablock sd76 - ellipsoid plot

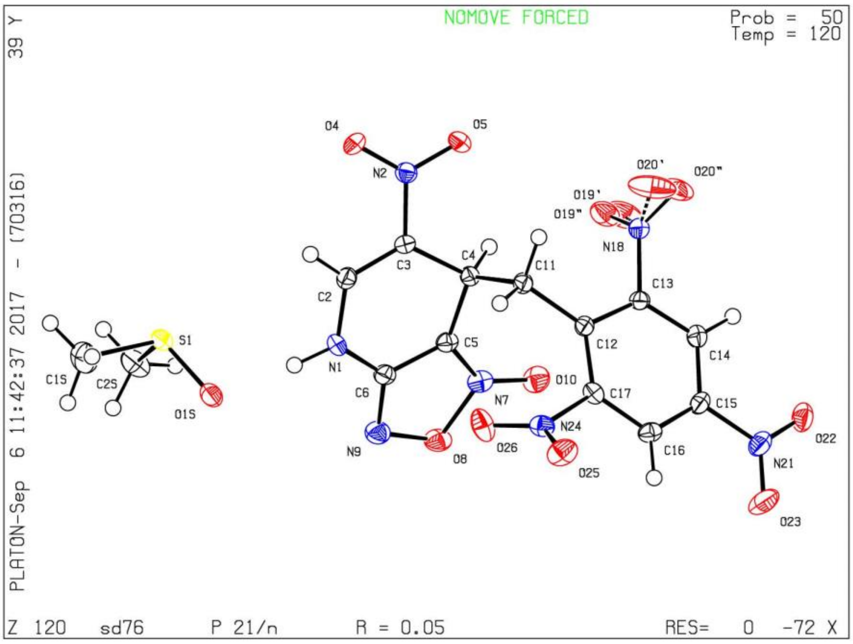

checkCIF/PLATON report

Structure factors have been supplied for datablock(s) sd77

THIS REPORT IS FOR GUIDANCE ONLY. IF USED AS PART OF A REVIEW PROCEDURE FOR PUBLICATION, IT SHOULD NOT REPLACE THE EXPERTISE OF AN EXPERIENCED CRYSTALLOGRAPHIC REFEREE.

No syntax errors found. CIF dictionary Interpreting this report

Datablock: sd77

```
Bond precision:   C-C = 0.0020 A          Wavelength=0.71073

Cell:             a=11.138(2)     b=12.765(3)          c=12.649(2)
                  alpha=90        beta=103.045(4)      gamma=90
Temperature:      120 K

                 Calculated                  Reported
Volume           1752.0(6)                   1752.1(6)
Space group      P 21/c                      P 21/c
Hall group       -P 2ybc                     -P 2ybc
Moiety formula   C11 H12 N4 O7, C3 H7 N O    C11 H12 N4 O7, C3 H7 N O
Sum formula      C14 H19 N5 O8               C14 H19 N5 O8
Mr               385.34                      385.34
Dx,g cm-3        1.461                       1.461
Z                4                           4
Mu (mm-1)        0.121                       0.121
F000             808.0                       808.0
F000'            808.47
h,k,lmax         15,17,17                    15,17,17
Nref             5111                        5112
Tmin,Tmax        0.961,0.969                 0.797,0.862
Tmin'            0.961

Correction method= # Reported T Limits: Tmin=0.797 Tmax=0.862
AbsCorr = MULTI-SCAN

Data completeness= 1.000           Theta(max)= 29.999

R(reflections)= 0.0425( 3840)      wR2(reflections)= 0.1153( 5112)

S = 1.032                  Npar= 256
```

The following ALERTS were generated. Each ALERT has the format
test-name_ALERT_alert-type_alert-level.
Click on the hyperlinks for more details of the test.

Alert level C

```
PLAT230_ALERT_2_C Hirshfeld Test Diff for    O10    --  N7      ..        5.3 s.u.
```

Alert level G

```
PLAT395_ALERT_2_G Deviating  X-O-Y  Angle from 120 Deg for  O8          107.7 Degree
PLAT432_ALERT_2_G Short  Inter X...Y Contact  O8      ..  C1S     ..     3.00 Ang.
PLAT432_ALERT_2_G Short  Inter X...Y Contact  O10     ..  C14     ..     2.86 Ang.
PLAT720_ALERT_4_G Number of Unusual/Non-Standard Labels ..........          7 Note
PLAT793_ALERT_4_G The Model has Chirality at C4      (Centro SPGR)          R Verify
PLAT960_ALERT_3_G Number of Intensities with I  <   - 2*sig(I) ...         14 Check
PLAT978_ALERT_2_G Number C-C Bonds with Positive Residual Density.         11 Info
```

```
   0 ALERT level A = Most likely a serious problem - resolve or explain
   0 ALERT level B = A potentially serious problem, consider carefully
   1 ALERT level C = Check. Ensure it is not caused by an omission or oversight
   7 ALERT level G = General information/check it is not something unexpected

   0 ALERT type 1 CIF construction/syntax error, inconsistent or missing data
   5 ALERT type 2 Indicator that the structure model may be wrong or deficient
   1 ALERT type 3 Indicator that the structure quality may be low
   2 ALERT type 4 Improvement, methodology, query or suggestion
   0 ALERT type 5 Informative message, check
```

It is advisable to attempt to resolve as many as possible of the alerts in all categories. Often the minor alerts point to easily fixed oversights, errors and omissions in your CIF or refinement strategy, so attention to these fine details can be worthwhile. In order to resolve some of the more serious problems it may be necessary to carry out additional measurements or structure refinements. However, the purpose of your study may justify the reported deviations and the more serious of these should normally be commented upon in the discussion or experimental section of a paper or in the "special_details" fields of the CIF. checkCIF was carefully designed to identify outliers and unusual parameters, but every test has its limitations and alerts that are not important in a particular case may appear. Conversely, the absence of alerts does not guarantee there are no aspects of the results needing attention. It is up to the individual to critically assess their own results and, if necessary, seek expert advice.

Publication of your CIF in IUCr journals

A basic structural check has been run on your CIF. These basic checks will be run on all CIFs submitted for publication in IUCr journals (*Acta Crystallographica, Journal of Applied Crystallography, Journal of Synchrotron Radiation*); however, if you intend to submit to *Acta Crystallographica Section C* or *E* or *IUCrData*, you should make sure that full publication checks are run on the final version of your CIF prior to submission.

Publication of your CIF in other journals

Please refer to the *Notes for Authors* of the relevant journal for any special instructions relating to CIF submission.

PLATON version of 13/08/2017; check.def file version of 27/07/2017

Datablock sd77 - ellipsoid plot

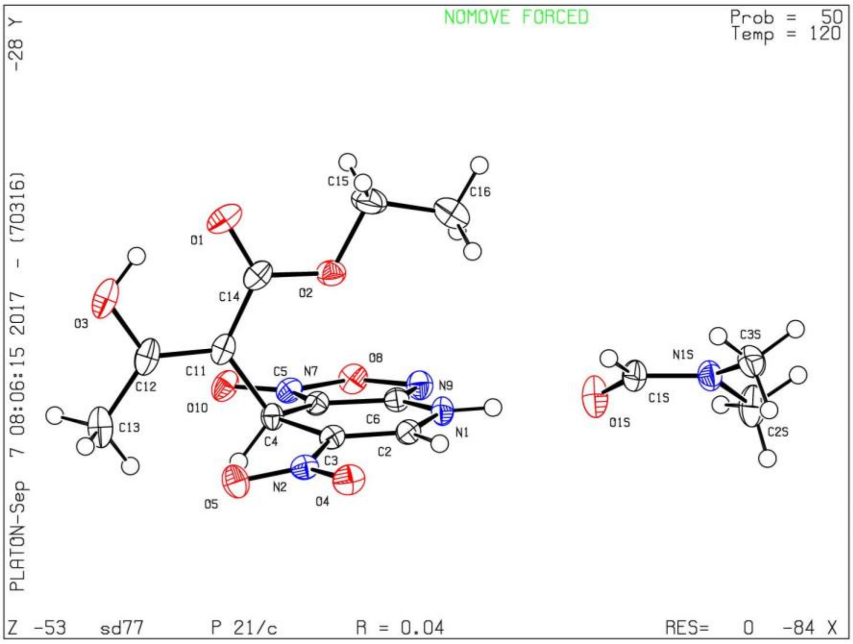

Supplement: File 1 — Experimental section, NMR spectra, HRMS and X-ray analysis data. [file Beilstein_J_Org_Chem-13-2854-s001.pdf]
